# HMGR Modulates Strawberry Fruit Coloration and Aroma Through Regulating Terpenoid and Anthocyanin Pathways

**DOI:** 10.3390/foods14071199

**Published:** 2025-03-29

**Authors:** Ting Zheng, Lingzhu Wei, Jiang Xiang, Jiang Wu, Jianhui Cheng

**Affiliations:** Institute of Horticulture, Zhejiang Academy of Agricultural Sciences, Hangzhou 310021, China; zhengting12309@163.com (T.Z.); weilingzhu@zaas.ac.cn (L.W.); xiangjiang717@163.com (J.X.); wujiang@zaas.ac.cn (J.W.)

**Keywords:** strawberry, HMGR, anthocyanins, aroma

## Abstract

HMGR is a crucial enzyme in the biosynthesis of terpenoids. We cloned *FaHMGR* and found that *FaHMGR* expression in fruit was significantly higher than other tissues, especially during the coloring stage. Suppression of *FaHMGR* (FaHMGRR) promoted coloration by increasing anthocyanin content and produced five new components. In contrast, *FaHMGR* overexpression (FaHMGROE) downregulated most anthocyanin genes and reduced hexanoic acid methyl ester and linalool contents, thereby inhibiting coloring. Transcriptomic and metabolomic analyses showed that DEGs in HMGROE vs. HMGRC (*pCAMBIA1302* empty vector transformant serving as a control) were significantly enriched in phenylpropanoid biosynthesis pathway and pathways related to terpenoid metabolism and MeJA, suggesting MeJA as a potential mediator of HMGR’s influence on terpenoid pathways. Additionally, DEGs in HMGRR vs. HMGRC were enriched in anthocyanin biosynthesis, particularly keracyanin and pelargonidin, which may explain the promoted coloration observed in HMGRR. WGCNA analysis identified five module genes with distinct expression patterns in HMGRR and HMGROE, including ERF118 and WRKY12, which may impact fruit quality by regulating HMGR activity.

## 1. Introduction

As the most abundant class of secondary metabolites in plants, terpenoids play an important role in plant growth and development, environmental adaptation, and fruit quality attributes. As a globally popular berry crop, strawberries (*Fragaria ananassa* (Weston) Duch.) are well-recognized for their distinctive flavor and nutritional benefits. In the French strawberry cultivar Falandi, nine sesquiterpenes and three triterpenes have been identified. Terpenes are important components of volatile organic compounds (VOCs). Although they constitute only 0.001–0.01% of strawberry weight, these compounds are essential to the fruit’s favorable flavor, with small changes greatly impacting taste [1]. Strawberry fruits produce a favorable aroma, with terpenoids significantly contributing to their aroma profile. Key terpenoids in strawberries include linalool, nerol, menthol, α-pinene, β-myrcene, α-terpineol, and β-phellandrene, which vary across varieties and developmental stages [2]. Among them, linalool and nerol are two major aroma components in strawberries; however, their contents differ significantly between wild (*Fragaria × vesca*) and cultivated strawberries (*Fragaria × ananassa*). The terpenoid synthase gene *FaNES1*, which is highly expressed in cultivated strawberries, catalyzes the conversion of geranyl diphosphate (GPP) and farnesyl diphosphate (FPP) into linalool and nerol [3]. A study by Fan et al. [4] identified grape flavor genes and their regulatory elements using multi-omics. This research implies that the distal region of strawberry Chromosome 3C may be related to the production of multiple terpenes, with *FaNES1* significantly correlated with the biosynthesis of linalool, β-myrcene, α-pinene, (E)-β-farnesene, and nerol.

Studies on the disease resistance mechanisms in strawberries suggest terpenes as one of the three crucial metabolites involved in strawberry defense [5]. Xu et al. [6] demonstrated that UV-C exposure induces monoterpene production in strawberry leaves, which interacts with abscisic acid (ABA) to enhance pathogen resistance. Additionally, terpinen-4-ol improves resistance against *Botrytis cinerea* and soft rot by activating the phenylpropanoid metabolic pathway in strawberry fruits [7]. Triterpene accumulation has also been linked to differential resistance across strawberry varieties exposed to nanoplastics [8]. Moreover, rapid induction of terpenoid synthesis may enhance anthracnose resistance in certain wild strawberry species [9].

Terpenoids are primarily synthesized in plants through two pathways: the mevalonate (MVA) pathway in the cytoplasm and the 2-C-methyl-D-erythritol-4-phosphate (MEP) pathway in the plastids [10]. In the MVA pathway, 3-hydroxy-3-methylglutaryl CoA reductase (HMGR) is the first rate-limiting enzyme and acts as a central regulator in the biosynthesis of cytoplasmic terpenoids [11]. Studies have demonstrated a positive correlation between HMGR activity and the production of diverse terpenoids across virtually all plant species [12,13,14,15]. Upregulation of HMGR and TPS genes has been shown to enhance terpene levels in roses [16]. Terpenoids influenced by HMGR include beneficial compounds such as sterols, rubber, resin, and saponins [17,18,19,20], which improve plant adaptability to adverse environmental conditions [21,22]. HMGR also directly affects the synthesis of sesquiterpene and triterpene aromas and accelerates the accumulation of tetraterpenoid carotenoids in *Escherichia coli* (*E. coli*). [23,24,25]. In addition, HMGR also participates in the regulation of anthocyanin accumulation in plants by affecting hormone accumulation and signal transduction. Downregulation of the HMGR gene can positively induce the expression of a variety of hormones and affect the accumulation of anthocyanins [19,24,26]. However, the association between HMGR and quality traits, such as fruit aroma and color, remains poorly understood [26], with no reported studies in strawberries. Given the significance of HMGR in strawberry fruit development and ripening, we cloned *FaHMGR* in strawberries and examined its expression across various tissues and developmental stages. Moreover, by integrating transcriptomic and metabolomic analyses with transient gene expression, we assessed the effect of *FaHMGR* on strawberry fruit quality attributes.

## 2. Materials and Methods

### 2.1. Plant Materials

Experiments were conducted at the Yangdu Experimental Vineyard, the Zhejiang Academy of Agricultural Science (E 120°24′, N 30°26′), Jiaxing, China. The strawberry plants used in this study were *Fragaria × ananassa* cv. Benihoppe. Young roots, stems, leaves, petals, and pistils were collected during flowering period, and the fruits were collected at different developmental stages, individually rapidly frozen in liquid nitrogen, and stored at −80 °C for the determination of *HMGR* gene expression.

### 2.2. RNA Extraction, cDNA Synthesis, and qRT-PCR

Total RNA was extracted from 0.1 g of each tissue material using the cetyltrimethylammonium bromide (CTAB) method with the FastPure Plant Total RNA Isolation Kit (Vazyme, Nanjing, China). cDNA was synthesized using a HifairII^®^ 1st Strand cDNA Synthesis SuperMix (Yeasen, Shanghai, China). The qRT-PCR reaction consisted of 5 μL SYBR Premix Ex Taq™ (Takara, Beijing, China), 0.3 μL of each primer (10 μM), 2 μL cDNA, and 2.4 μL RNase-free water, for a total volume of 10 μL. Reactions were performed on a LightCycler 1.5 instrument (Roche, Baden, Germany), starting with the preliminary step at 95 °C for 30 s, followed by 35 cycles of 95 °C for 5 s and 58 °C for 35 s [27]. The relative gene expression was calculated using the 2^−ΔCt^ method [10]. Experiments were conducted with three biological and three technical replicates. Gene-specific primers are listed in Table S1.

### 2.3. Determination of Hormone Contents

The contents of the hormones auxin indole-3-acetic acid (IAA), ABA, gibberellin (GA_3_), zeatin riboside (ZR), and brassinosteroids (BRs) were determined using an enzyme-linked immunosorbent assay (ELISA), following the instruction by Wang et al. [28]. For hormone extraction, 0.5 g of strawberry fruits were ground in an ice bath with 2 mL of extract solution (80% methanol containing 1 mmol·L^−1^ BHT). The resulting mixture was diluted to 10 mL, incubated at 4 °C for 4 h, and then centrifuged at 3500 rpm for 8 min. The supernatant was collected and extracted by C-18 solid phase extraction. The eluted sample was transferred to a 10 mL centrifuge tube and freeze-dried before being dissolved in 2 mL of buffer.

### 2.4. Transient Expression Levels of FaHMGR and FaHMGRi in Strawberries

Full-length coding sequences of *FaHMGR* and *FaHMGRi* (see Table S2) were amplified from the cDNA of ‘Benihoppe’ strawberries and cloned into the *pCAMBIA1302* vector carrying a 35S promoter (abbreviated as *p35S*) to generate constructs *p35S-FaHMGR-GFP* and *p35S-FaHMGRi-GFP*. For transient expression, these two plasmids and *pCAMBIA1302* empty vector were individually mobilized into *Agrobacteria* (strain *EHA105*), which were then used to transform strawberries at the green stage [29], generating the transgenic lines FaHMGROE and FaHMGRR, with the *pCAMBIA1302* empty vector transformant serving as a control (CK, FaHMGRC). Following confirmation of the expression levels of *FaHMGR-GFP* and *FaHMGRi-GFP* by qRT-PCR (gene specific primers are listed in Table S2), the anthocyanin content and aroma components were determined.

### 2.5. Determination of Anthocyanin and Chlorophyll Content

Chlorophyll content was determined using spectrophotometry. Total anthocyanins were extracted using a methanol–HCl method. Samples of 0.1 g were submerged and incubated overnight in 5 mL of methanol containing 0.1% (*v*/*v*) HCl in the dark at room temperature. Anthocyanin content was measured using the PH differential method, with absorbance at 520 and 700 nm recorded using a UV-2550 spectrophotometer (Shimadzu, Kyoto, Japan). The anthocyanin components were determined using liquid chromatography-mass spectrometer (LC-MS), and the peak area was measured to quantify each component [30].

### 2.6. Determination of the Aroma Components 

Aroma components in transgenic strawberries FaHMGROE, FaHMGRR, and FaHMGRC were analyzed using gas chromatography-mass spectrometry (GC-MS). According to the method described by Zheng et al. [30], samples (3 g) were ground, transferred to a 20 mL headspace bottle, and mixed with three milliliters of saturated NaCl solution and 2 μL 3-nonanone (internal standard). The samples were analyzed with a gas chromatograph (TRACE 1310, Thermo Scientific, Shanghai, China) coupled to a triple quadrupole mass spectrometer (TSQ 9000, Thermo Scientific, Shanghai, China). The column temperature was programmed as follows: the initial temperature was set at 50 °C for 6 min and then increased to 250 °C at a rate of 6 °C min^−1^, and held for 3 min. The MS conditions were as follows: EI mode at voltage 70 eV; ion source temperature at 230 °C; a scanning rate of 2.88 scan·s^−1^; and a detection range of 29–540 m·z^−1^, with helium as the carrier gas at a flow rate of 1.0 mL min^−1^.

### 2.7. Transcriptomic Analysis

Library preparation and transcriptome sequencing of FaHMGROE, FaHMGRR, and FaHMGRC strawberries were conducted by the Beijing Novogene Technology Corporation (Beijing, China). Differentially expressed gene (DEG) analysis was performed using the DESeq R package (1.18.0) with the following criteria: false discovery rate (FDR) < 0.05, |fold change| ≥ 2, and adjusted *p*-value < 0.05 [31]. Principal component analysis (PCA) was performed with the gmodels R package. Gene Ontology (GO) enrichment and Kyoto Encyclopedia of Genes and Genomes (KEGG) pathway analyses were performed with the GOseq R 3.12 software package and KOBAS 3.0 software, respectively [32]. A weighted gene correlation network (WGCNA) was constructed based on a hierarchical clustering tree. All treatments included three biological replicates. To validate transcriptomic data, ten DEGs were selected for confirmation via qRT-PCR.

### 2.8. Metabolomic Profile Detection and Analysis

For metabolome sequencing, fruits of strawberries FaHMGROE, FaHMGRR, and FaHMGRC were individually grounded in liquid nitrogen, and each homogenate was resuspended in pre-chilled 80% methanol and 0.1% formic acid and well vortexed before incubation. The samples were then centrifuged, diluted, and filtered before injection into the LC-MS/MS system. LC-MS/MS analysis was performed using a Vanquish UHPLC system (Thermo Scientific, Shanghai, China) coupled with an Orbitrap Q Exactive HF-X mass spectrometer (Thermo Fisher). Raw data were processed using Compound Finder 3.0 (CD 3.0, Thermo Fisher). Peaks were matched with the mzCloud (https://www.mzcloud.org/, accessed on 20 March 2022) and ChemSpider (http://www.chemspider.com/, accessed on 20 March 2022) databases to obtain accurate qualitative results, with areas measured for quantitative analysis. The statistical analyses were conducted using R (R version R-3.4.3), Python (Python 2.7.6 version), and CentOS (CentOS release 6.6). Positive ion mode (POS) and negative ion mode (NEG) were both used to detect metabolites. The quality control (QC) sample was included to assess system stability, and a blank sample was used to eliminate background ions.

### 2.9. Statistical Analysis

All data (with at least three replications, *n* = 3) are presented as means with standard errors of the means (SEMs). Mean ± SEM values for each treatment were calculated using Microsoft Excel (Microsoft Corporation, Albuquerque, NM, USA). Statistical analysis of variance (ANOVA) and Duncan’s multiple range test (*p* < 0.05) were performed using SPSS 17.0 (SPSS, Inc., Chicago, IL, USA). Figures were generated using Origin Pro 9 (Origin Inc., Northampton, MA, USA).

## 3. Results

### 3.1. Expression Characteristics of FaHMGR

**Figure 1:**
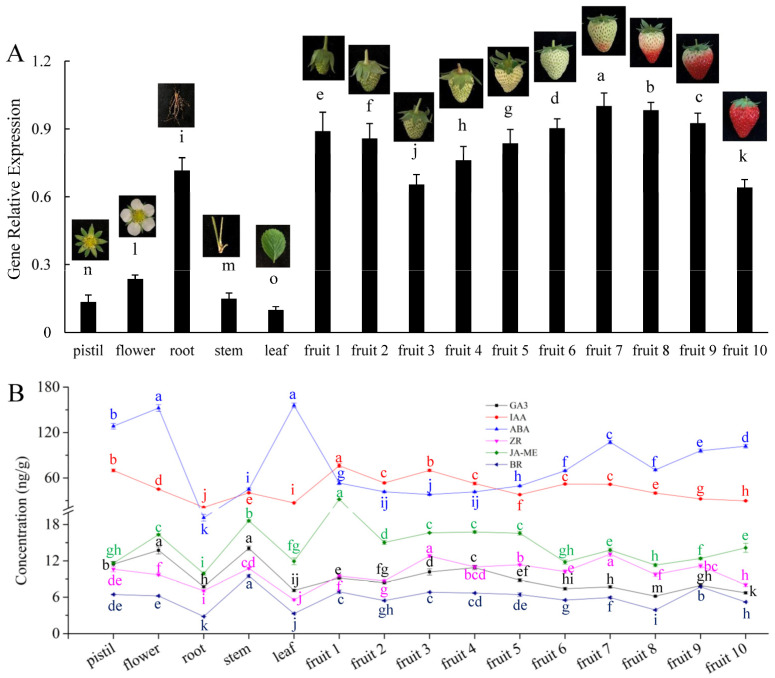
Expression of *FaHMGR* (**A**) and hormone levels (**B**) in various tissues and fruits at different developmental stages. Different letters represent significant differences between groups.

To clarify the patterns of FaHMGR accumulation in strawberries, we examined its expression across various tissues and observed significant variation, with the highest level found in fruit tissue, followed by root, flower, stem, pistil, and leaf in descending order (Figure 1A). During fruit development, *FaHMGR* expression gradually decreased during the fruit enlargement phase (fruit 1 to fruit 3), then began to increase steadily during the fruit whitening phase (fruit 4 to fruit 6), peaking at the onset of fruit coloring (fruit7). The transcription of *FaHMGR* then decreased as the fruits turned red, reaching its lowest level at fruit mature stage (fruit 10).

Analyses of six hormones across different tissues and at various stages of fruit development revealed that IAA and ABA were significantly more abundant than the other hormones, including GA3, ZR, JA-ME, and BRs. JA-ME levels were slightly lower than those of IAA and ABA, while BR levels were the lowest across examined tissues (Figure 1B). Notably, hormone levels in roots were generally lower than in other tissues, contrasting with the high expression of *HMGR* in this tissue. At the onset of fruit coloring (fruit 7), levels of GA_3_, ABA, ZR, JA-ME, and BRs all increased to varying extents and then decreased as fruit coloration progressed (fruit 8).

### 3.2. Effects of Transient FaHMGR and FaHMGRi Expression on Strawberry Coloration and Aroma

**Figure 2:**
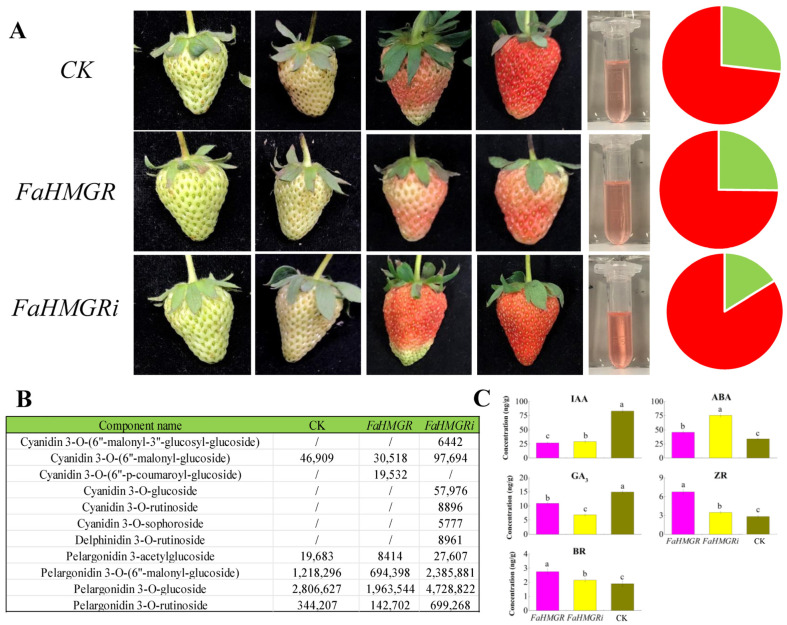
Influence of *FaHMGR* expression on strawberry fruit development. (**A**) Fruit development in strawberries transiently overexpressing *FaHMGR* (FaHMGROE) and *FaHMGRi* (FaHMGRR), using the *pCAMBIA1302* empty vector transformant as a control (CK). The pie chart illustrates the relative chlorophyll (green) and anthocyanin (red) contents in strawberries. (**B**) Composition of anthocyanins in strawberries with varying FaHMGR levels. Data in the table represent the peak area. (**C**) Impact of *FaHMGR* overexpression and suppression on phytohormone levels in strawberries treated as in (**A**). Different letters represent significant differences between groups.

Overexpression of *HMGR* in strawberries inhibited fruit coloration and reduced anthocyanin content, where opposite phenomena were observed when *HMGR* was suppressed, with a 3.33 mg·g^−1^ increase in anthocyanin content compared to the control (Figure 2A). Analysis of anthocyanin components revealed that cyanidin 3-O-(6″-malonyl-3″-glucosyl-glucoside), cyanidin 3-O-glucoside, cyanidin 3-O-rutinoside, cyanidin 3-O-sophoroside, and delphinidin 3-O-rutinoside were present in FaHMGRR strawberries but absent in FaHMGRC and FaHMGROE (Figure 2B). Additionally, the concentrations of other anthocyanin components in FaHMGRR were significantly higher than in both FaHMGRC and FaHMGROE strawberries. Cyanidin 3-O-(6″-p-coumaroyl-glucoside) was only detected in FaHMGROE. Furthermore, the content of ABA in FaHMGRR was significantly higher than in both FaHMGROE and FaHMGRC (Figure 2C). Conversely, the concentrations of BRs, ZR, and GA_3_ were significantly elevated in FaHMGROE plants compared to FaHMGRR strawberries.

**Figure 3:**
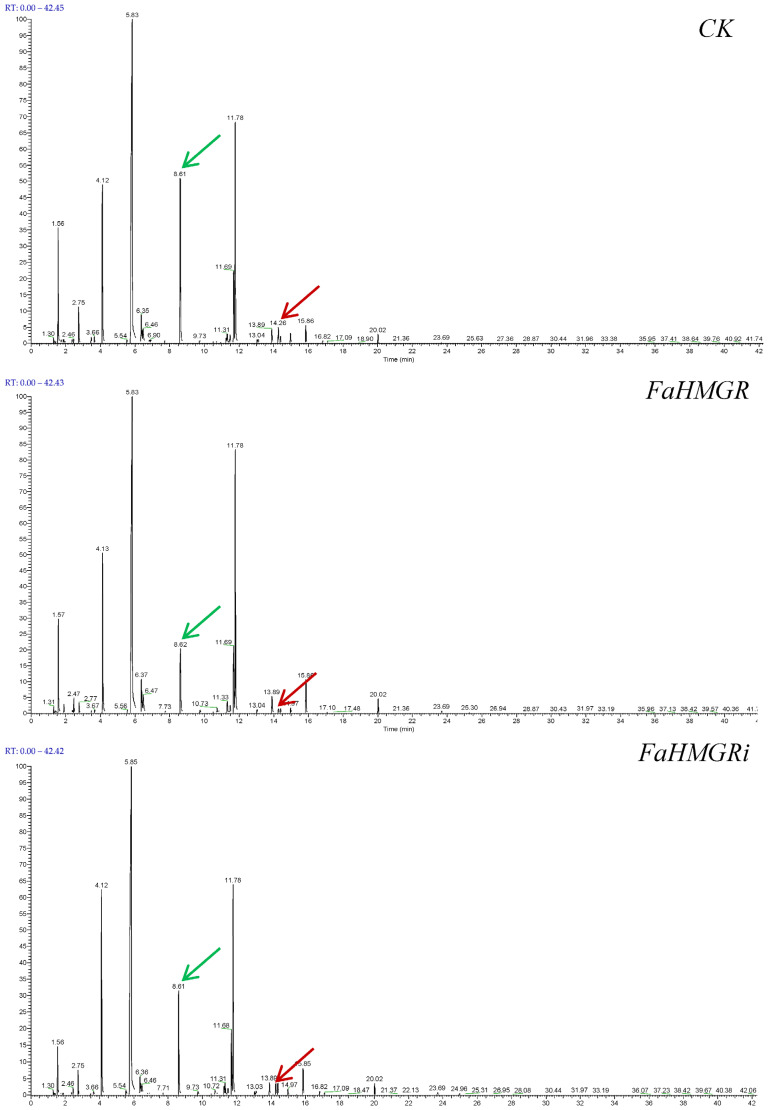
Impact of *FaHMGR* overexpression and suppression on fruit aroma components in strawberries. The red arrow indicates linalool, which decreased after both *FaHMGR* overexpression and suppression; and the green indicates methyl hexanoate, another main aroma component, which also showed a decrease in response to changes in *FaHMGR* expression. (The specific data are presented in Table 1).

The type and content of aroma compounds are crucial in determining strawberry fruit flavors and distinguishing strawberry varieties. In this study, a total of 93, 84, and 120 aroma compounds—including esters, ketones, aldehydes, alcohols, terpenes, and minor amounts of furans, phenols, and acids—were detected in FaHMGROE, FaHMGRR, and FaHMGRC strawberries, respectively (Figure 3, Table 1). Esters were found to be the dominant aroma components in wild-type strawberries, with hexanoic acid and its methyl ester accounting for 18.96%. Their concentrations decreased in FaHMGROE and FaHMGRR strawberries. Another major component, (Z)-hex-2-enyl acetate, decreased in FaHMGRR strawberries but remained unchanged in FaHMGROE. Linalool, the primary terpene aroma in strawberries, had a content of 0.62% in CK, which decreased to 0.13% and 0.37% in FaHMGROE and FaHMGRR strawberries, respectively. Additionally, other detected terpenes included D-limonene (0.16%) in CK and α-pinene (0.03%), β-pinene (0.05%), α-terpineol (0.01%), and caryophyllene (0.01%) in FaHMGRR strawberries.

**Figure 4:**
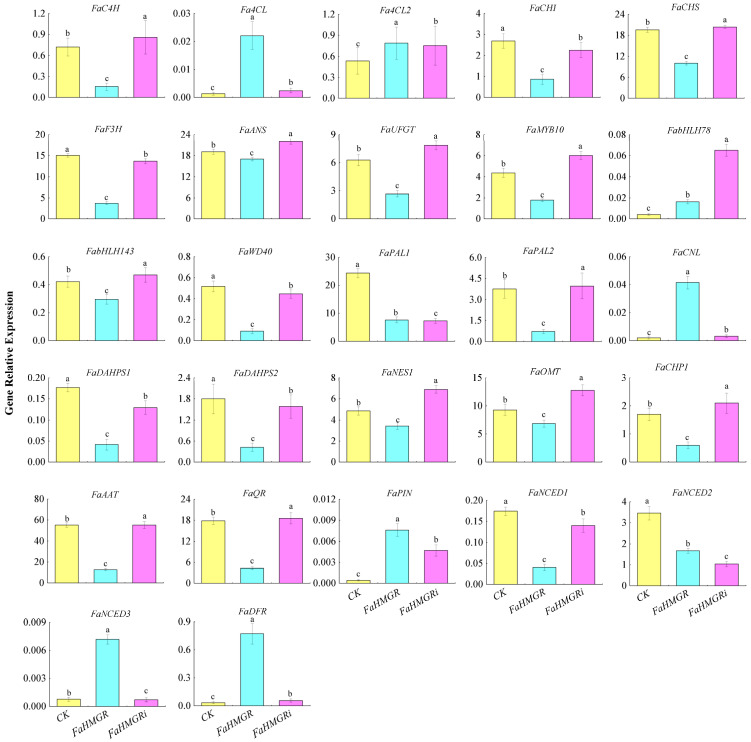
Effects of changes in FaHMGR levels on the expression of genes associated with anthocyanin and aroma metabolic pathways following transient overexpression of *FaHMGR* or suppression via *FaHMGRi* in strawberries. The pCAMBIA1302 empty vector was used as a control. Different letters represent significant differences between groups.

The expression levels of genes involved in anthocyanin and aroma formation were assessed in FaHMGROE, FaHMGRR, and FaHMGRC strawberries. The results showed that the expression of key anthocyanin metabolic pathway genes, including *FaC4H*, *FaCHI*, *FaCHS*, *FaF3H*, *FaANS*, *FaUFGT*, *FaMYB10*, *FabHLH143*, *FaWD40*, and *FaPAL2*, was lowest in FaHMGROE strawberries, whereas *Fa4CL*, *Fa4CL2*, and *FaDFR* increased. In contrast, in FaHMGRR fruits, levels of *FaC4H*, *FaCHS*, *FaANS*, *FaUFGT*, *FaMYB10*, *FabHLH143*, *FaWD40*, and *FaPAL2* were higher than in both CK and FaHMGROE strawberries (Figure 4). This result suggests a negative correlation between FaHMGR level and the anthocyanin accumulation. For aroma synthesis genes, *FaCNL*, *FaPIN*, and *FaNCED3* exhibited the highest expression in FaHMGROE fruits, while *FaDAHPS1*, *FaDAHPS2*, *FaNES1*, *FaOMT*, *FaCHP1*, *FaAAT*, *FaQR*, and *FaNCED1* showed the lowest expression. When *FaHMGR* was inhibited, genes *FaNES1*, *FaOMT*, *FaCHP1*, *FaAAT*, *FaQR*, and *FaNCED1* were significantly upregulated.

### 3.3. Manipulating FaHMGR Expression Caused Changes in Strawberry Transcription and Metabolism

To determine the transcriptional and metabolic regulatory effects of *FaHMGR* on strawberries, RNA sequencing (RNA-Seq) and metabolic profiling were performed on the strawberries with transient overexpression or suppression of *FaHMGR* (HMGROE, HMGRR), with the empty vector transformed plants as the control (HMGRC). RNA sequencing generated approximately 6.6 GB of data and an average of 44,377,805 clean reads (Table S3). Mapping to the strawberry genome database yielded a high coverage of 94.77%, suggesting effective detection of transcriptional changes due to overexpression alteration of *FaHMGR* in strawberries. To validate RNA-Seq reliability, 10 DEGs were randomly selected for qRT-PCR, which revealed consistent expression patterns of these genes with the sequencing results (Figure S1).

**Figure 5:**
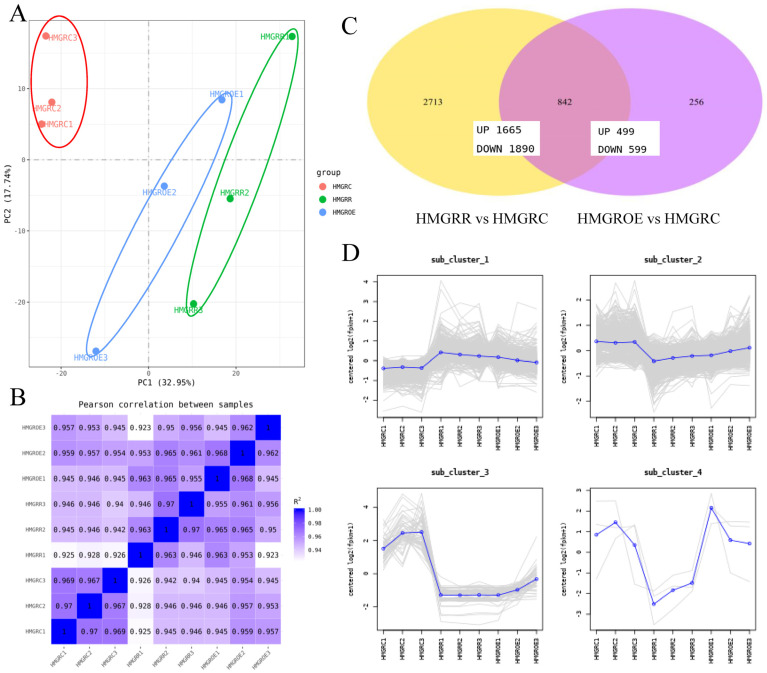
Differentially expressed genes (DEGs) in strawberries after the overexpression and suppression of *FaHMGR* in strawberry fruits. (**A**). Principal component analysis (PCA) scatter plot of transcriptomic profiles from different samples. (**B**). Correlation coefficient graph showing the relationships among all samples, demonstrating the degree of similarity in gene expression profiles. (**C**). Venn diagram illustrating the number of DEGs in comparison pairs HMGROE vs. HMGRC and HMGRR vs. HMGRC. (**D**). Expression patterns of DEGs. The *Y*-axis represents the *p*-value and the *X*-axis represents samples.

The PCA of the transcriptome data (Figure 5A) revealed that all samples were positioned along the positive axis of PC1, which explained 32.95% of the variance, indicating a strong association among HMGROE, HMGRR, and HMGRC. HMGROE and HMGRR overlapped along PC1, while minimal differences were observed among the three samples along PC2 (17.74%), with HMGRC positioned in the positive axis. As shown in Figure 5B, the correlation among triplicates for each treatment sample was high, exceeding 0.923. T1 showed slightly lower correlations (below 0.97) with the other two replicates. A total of 3811 DEGs were identified across all samples, with 2713 unique to the comparison pair HMGRR vs. HMGRC and 236 unique to HMGROE vs. HMGRC. There were more downregulated genes than upregulated ones in both comparison pairs (Figure 5C). Of these DEGs, 842 were common between HMGRR vs. HMGRC and HMGROE vs. HMGRC. All DEGs showed four distinct expression patterns and were thereby clustered into four groups comprising 1705, 2049, 54, and 3 genes. Most genes exhibited no significant differences between HMGROE and HMGRR but differed from HMGRC. In contrast, genes in cluster 4, including cold-regulated 413 plasma membrane protein 1 (*AtCOR413-PM1*), were expressed significantly differently between HMGROE and HMGRR (Figure 5D).

**Figure 6:**
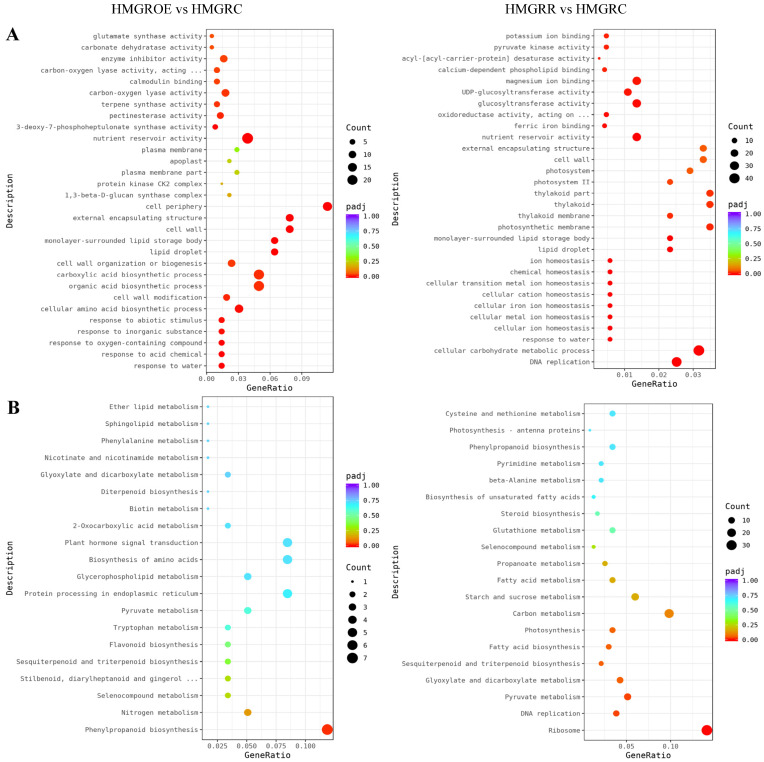
Gene Ontology (GO) categories (**A**) and Kyoto Encyclopedia of Genes and Genomes (KEGG) pathway analysis (**B**) of significantly enriched DEGs in the comparisons HMGRR vs. HMGRC and HMGROE vs. HMGRC.

GO and KEGG enrichment analyses of the DEGs in HMGRR vs. HMGRC and HMGROE vs. HMGRC were performed to identify the functional roles of the DEGs. GO analysis revealed that these DEGs in both the HMGROE vs. HMGRC and HMGRR vs. HMGRC comparisons showed significant enrichment in the item nutrient reservoir activity (Figure 6A, Table S4). KEGG analysis showed significant enrichment of the DEGs in the phenylpropanoid biosynthesis pathway for HMGROE vs. HMGRC and in the ribosome, DNA replication, and pyruvate metabolism pathways for the HMGRR vs. HMGRC comparison (Figure 6B, Table S4).

Furthermore, WGCNA divided the DEGs into 63 modules (Figure S2). Module–sample relationship analysis identified five modules indicated in Table S5 by the colors dark magenta, honeydew, tan, violet, and white, containing 82, 40, 269, 88, and 102 genes, respectively (Table S5). Genes in each module displayed distinct expression patterns in HMGRR and HMGROE strawberries (Table S5). In the violet module, gene expression was low in HMGRR and high in HMGROE, while the other four modules exhibited the opposite pattern. Notably, two abscisic acid receptors, *PYL11* and *PYL9*, were identified in the dark magenta and honeydew modules. The tan, violet, and white modules contained several enzymes involved in sugar and acid synthesis pathways. Additionally, transcription factors ERF118 and WRKY12 were found in the white module, suggesting that they may play an important role in *FaHMGR*-mediated effects on fruit quality.

**Figure 7:**
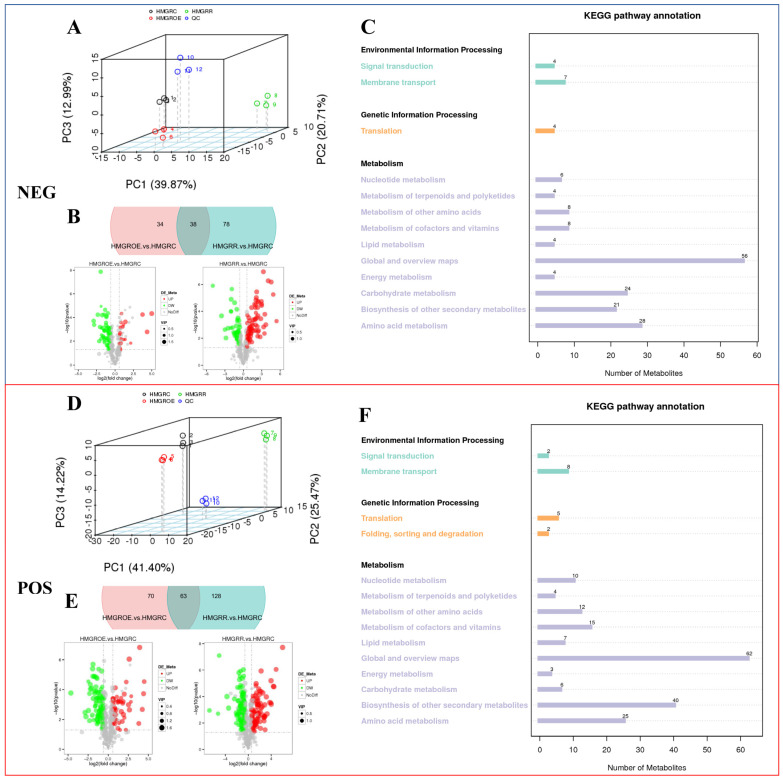
Differentially accumulated metabolites (DAMs) in negative and positive ion modes (NEG, POS) in strawberries after the overexpression and suppression of *FaHMGR* in fruits. (**A**,**D**). PCA analyses in NEG and POS. Quality control (QC) was an equivalent standard to evaluate the stability and signal response strength of the instrument during metabolite detection. (**B**,**E**). Venn diagrams and volcano maps illustrating the differential metabolites and their quantities. (**C**,**F**). Kyoto Encyclopedia of Genes and Genomes (KEGG) annotation of identified metabolites in samples.

By integrating database comparisons, we conducted qualitative and quantitative analyses of metabolites, identifying a total of 501 components in positive ion mode and 312 in negative ion mode (Table S6). Principal component analysis (PCA) was employed to evaluate the overall metabolic differences between samples (Figure 7A,D), revealing that HMGROE was more closely related to HMGRC than to HMGRR. In positive ion mode, 63 differential metabolites were common to both HMGROE vs. HMGRC and HMGRR vs. HMGRC. Additionally, 70 and 128 unique metabolites were identified in each pairwise comparison, respectively (Figure 7B,E). In the negative ion mode, 30 differential metabolites were shared in the two pairs, with 34 and 70 metabolites uniquely present in each. Further analysis showed that in HMGROE vs. HMGRC, 20 differential metabolites were upregulated and 52 were downregulated in negative ion mode, while 133 were upregulated and 131 were downregulated in positive ion mode. For HMGRR vs. HMGRC, 116 differential metabolites were upregulated and 115 were downregulated in negative ion mode, while 191 were upregulated and 189 were downregulated in positive ion mode. KEGG pathway analysis indicated that most metabolites mapped to the item “Global and overview maps”, with four specific substances associated with the “Metabolism of terpenoids and polyketides” pathway in both negative and positive ion modes (Figure 7C,F).

KEGG analysis of differentially accumulated metabolites (DAMs) identified 30 enriched pathways in HMGROE vs. HMGRC, with significant enrichment in the phenylpropanoid biosynthesis pathway (*p* value = 0.029) (Table S7). It is worth noting that the DEGs were enriched in the diterpenoid biosynthesis pathway (*p* value = 0.228), indicating that *HMGR* overexpression caused DAMs to decrease and affected terpenoid metabolism. For HMGRR vs. HMGRC, 56 pathways were enriched, including ubiquinone and other terpenoid–quinone biosynthesis pathways (*p* value = 0.500), with specific metabolites such as shikonin linked to the HMGR metabolic pathway. This group was also enriched in the anthocyanin biosynthesis pathway (*p* value = 0.126), involving metabolites like keracyanin and pelargonidin, which is likely one reason for the enhanced coloration in strawberries under the HMGRR condition. Notably, both HMGROE vs. HMGRC and HMGRR vs. HMGRC were enriched in methyl jasmonate-related pathways, including alpha-linolenic acid metabolism and plant hormone signal transduction (*p* value = 0.500).

## 4. Discussion

### 4.1. Correlation Between HMGR Expression Levels and Strawberry Fruit Quality

Research on HMGR and its influence on fruit quality related to terpenoids remains limited, particularly concerning its effects on fruit color and aroma. The two terpenoid biosynthesis pathways MVA and MEP are not completely isolated, as transporters in the plastid membrane allow metabolite exchange, facilitating a ‘metabolic cross-talk’ [1]. HMGR, which typically catalyzes the conversion of 3-hydroxy-3-methyl-glutaryl-CoA to mevalonic acid in the MVA pathway, has been shown to influence color formation by affecting the MEP pathway and anthocyanin synthesis [10]; it also encodes functional proteins that accelerate carotenoid biosynthesis [23,25]. Consistently, Zheng et al. [10] found that the expression of *VvHMGRs* in yellow grape varieties was generally higher than in red varieties. HMGR positively regulates terpenoid metabolism, thereby affecting hormone synthesis and indirectly regulating anthocyanin accumulation [24]. Moreover, a truncated form of HMGR (tHMG) was found to increase endogenous sesquiterpenes level by 37-fold and phytoene by 100-fold [33]. Negative regulation of HMGR by phyB and HY5 via interaction with PIF3 may further affect anthocyanin synthesis [19]. In this study, strawberries overexpressing *FaHMGR* exhibited reduced coloration, along with downregulated expression of most anthocyanin pathway genes. Conversely, *HMGR* silencing by expressing *FaHMGRi* promoted strawberry coloration, with anthocyanin content increasing by 3.33 mg·g^−1^ compared to the control, aligning with findings by Zheng et al. [26] in grape studies.

Strawberry aroma contributes significantly to fruit quality, enhancing consumer appeal and potential health benefits. Strawberry aroma results from a mixture of more than 350 volatile compounds, making it one of the most complex fruit aromas [34]. Volatiles in strawberry aroma include esters, terpenoids, alkanes, alcohols, acids, aldehydes, and ketones, with terpenes providing the main floral notes [35]. The terpenoids responsible for strawberry aroma primarily consist of monoterpenes (C10), sesquiterpenes (C15), and triterpenes (C30). Monoterpenes were the only type detected in this study. The highest diversity of terpenes, especially monoterpenes, was observed in *FaHMGRi*-expressing strawberries. This finding suggests that inhibiting *HMGR* expression promotes monoterpene synthesis via the MEP pathway. However, this contrasts with studies in grapes, which indicated a positive correlation between *HMGR* expression and monoterpene accumulation. This inconsistency suggests species-specific differences in how *HMGR* influences fruit aroma composition [36].

Beyond color and aroma, HMGR expression also affects fruit size. HMGR impacts early fruit development by regulating MVA synthesis, a role confirmed in studies of melons and tomatoes. Moreover, reduced HMGR activity has been shown to disrupt normal plant development [37,38].

### 4.2. Key Factors Regulating HMGR Expression

Hormones are important regulators of terpenoid synthesis, functioning by influencing HMGR activity. Several studies have demonstrated that exogenous application of hormones, including ABA and indole-3-acetic acid (IAA), regulates HMGR activity, while CTK counteracts HMGR inhibitors to support cell growth [39,40,41,42]. Zheng et al. [26] found that BR treatment in Kyoho grapes inhibited *HMGR* expression and promoted fruit coloration. Here, our study showed significantly higher BR levels in FaHMGROE strawberries than in FaHMGRR.

Several key proteins interact with HMGR to regulate its activity, including Protein Phosphatase 2A (PP2A), sucrose nonfermenting-1 (SNF1)-related kinase 1 (SnRK1), HIGH STEROL ESTER 1 (HISE1), Phytoene synthases 1 (PSY1), GATA24, and PHYTOCHROME INTERACTING FACTOR 3 (PIF3). PP2A, a multi-stage regulator of plant HMGR, negatively impacts HMGR activity through post-translational regulation and is involved in signal transduction for hormones such as ABA, IAA, BRs, and ETH [40]. SnRK1 helps maintain energy homeostasis by post-translationally regulating HMGR and TPS5 [43]. Protein HISE1 participates in the downregulation of HMGR to prevent sterol overproduction in the endoplasmic reticulum, as evidenced by the loss-of-function mutant *hise1*, which displays elevated levels of HMGR and sterol-producing activity [44]. Additionally, tHMG1 complexes with ZmPSY1 and PaCRTI to significantly boost carotenoid accumulation in rice endosperm by enhancing MVA pathway flux and creating a metabolic sink for carotenoids [45]. In *Vitis vinifera*, twelve amino acid residues of VvGATA24, including Pro218B, directly bind to the GATA-box cis-element of *VvHMGR2* to mediate wax terpenoid biosynthesis [46].

### 4.3. Transcription Factors Related to Terpenoid Synthesis

Terpenoids encompass a diverse range of compounds with intricate metabolic regulation. The synthesis of plant terpenoids is a complex process involving various enzymes encoded by structural genes that catalyze the formation of multiple intermediate and final products in the terpenoid biosynthesis pathways. Regulatory genes are another crucial factor influencing terpenoid accumulation, forming complexes either individually or through interactions, and specifically binding to cis-elements in the promoters of structural genes. These regulatory complexes modulate terpenoid synthesis by upregulating or downregulating the expression of structural genes.

Transcription factors play a critical role in regulating multiple key genes involved in terpenoid metabolism [47]. Identifying these transcription factors contributes to our understanding of plant terpenoid synthetic biology. Six transcription factor families are currently known to be involved in terpenoid production, including AP2/ERF, bHLH, MYB, NAC, WRKY, and bZIP [6,46,48,49]. For example, AaERF1 and AaERF2 bind to CBF2 and RAA motifs in the promoters of ADS and CYP-71AV1 to regulate artemisinin synthesis [50]; in *Citrus sinensis*, citERF71 activates CitTPS16 geraniol synthase expression by binding to the CitTPS16 promoter [51]. WRKY regulates plant physiological and biochemical processes by modulating signaling pathways such as SA, ABA, JA, and ETH, thereby participating in plant growth, development, and stress responses. In *Catharanthus roseus*, *Gossypium arboretum*, and *Artemisia annua*, *WRKY1* has been shown to regulate monoterpene and sesquiterpene synthesis. For instance, CrWRKY1 inhibits CrMYC2 transcription to suppress monoterpene synthase, thereby regulating monoterpene production [52]. *GaWRKY1* promotes sesquiterpene synthesis in cotton by upregulating *CAD1-A* expression [53]; AaWRKY1, induced by MeJA, binds to the W-box cis-acting element of the ADS promoter to enhance artemisinin synthesis in *Artemisia annua* [54].

Research on HMGR-related transcription factors remains limited, and current knowledge primarily derives from *Arabidopsis thaliana*. Studies indicate that HY5 and PIF3 act antagonistically to regulate *HMGR* expression and sterol biosynthesis by physically binding to the AtHMGR2 promoter [55]. In this study, WGCNA analysis suggests that ERF118 and WRKY12 may act as transcription factors that modulate HMGR-related pathways affecting fruit quality.

## 5. Conclusions

**Figure 8:**
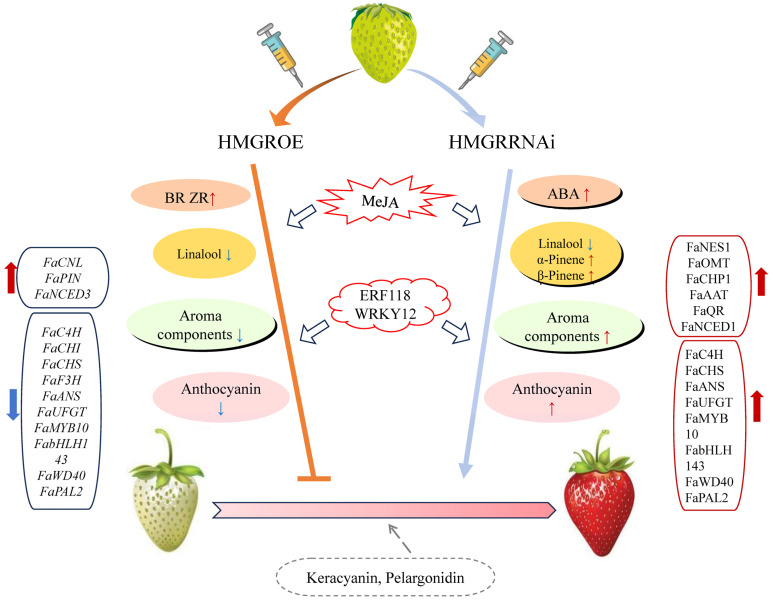
Proposed model illustrating the impact of varying *HMGR* expression levels on transcriptional changes and metabolite profiles linked to strawberry fruit development and aroma production.

In conclusion, this study offers new insights into the role of HMGR in influencing strawberry fruit coloration and aroma composition (Figure 8). HMGR regulates both the terpenoid and anthocyanin pathways, with its overexpression inhibiting fruit coloration, downregulating most anthocyanin pathway genes, and reducing aroma content. Conversely, suppression of HMGR promoted coloration and led to the identification of five new anthocyanin components. Our work also suggests MeJA as a potential mediator of HMGR’s influence on terpenoid pathways. Additionally, transcription factors ERF118 and WRKY12 potentially regulate HMGR to affect fruit quality. Overall, our findings contribute to the current understanding of terpenoid metabolism and strawberry fruit quality regulation.

## Figures and Tables

**Table 1:** Aroma components in the fruits of *FaHMGR* overexpressing and suppressing strawberry.

Component Name	Retention Time	Reference m/z	BP Area		Content (%)	BP Height	TIC	Formula (mol ion)	CAS No.	SI	RSI	Selected Column Type	Library Name	Sample Name
Acetaldehyde	1.371	44.055	10,228,276	3,136,768,594	0.33%	3,100,333	5,375,014	C2H4O	75-07-0	958	958	SemiStandardNonPolar	mainlib	CK
Oxirane, methyl-, (S)-	1.499	43.044	788,634	3,136,768,594	0.03%	421,871	1,257,665	C3H6O	16088-62-3	749	749	SemiStandardNonPolar	mainlib	CK
Borane-methyl sulfide complex	1.552	62.053	24,302,374	3,136,768,594	0.77%	11,909,122	42,681,680	C2H9BS	13292-87-0	883	883	SemiStandardNonPolar	mainlib	CK
Acetic acid, methyl ester	1.566	43.044	419,440,351	3,136,768,594	13.37%	182,866,394	265,690,168	C3H6O2	79-20-9	945	945	SemiStandardNonPolar	mainlib	CK
2,3-Butanedione	1.747	43.044	24,367,025	3,136,768,594	0.78%	6,939,979	9,434,886	C4H6O2	431-03-8	938	938	SemiStandardNonPolar	mainlib	CK
Ethyl Acetate	1.871	43.044	17,699,284	3,136,768,594	0.56%	6,485,836	9,925,005	C4H8O2	141-78-6	917	917	SemiStandardNonPolar	mainlib	CK
Trichloromethane	1.895	83.059	5,174,400	3,136,768,594	0.16%	2,556,596	9,359,119	CHCl3	67-66-3	882	882	SemiStandardNonPolar	mainlib	CK
Methyl propionate	1.965	57.062	4,721,554	3,136,768,594	0.15%	1,754,794	3,203,729	C4H8O2	554-12-1	938	938	SemiStandardNonPolar	mainlib	CK
Isopropyl acetate	2.173	43.044	3,258,741	3,136,768,594	0.10%	1,124,586	1,737,864	C5H10O2	108-21-4	873	880	SemiStandardNonPolar	mainlib	CK
tert-Butyl N-(tert-butoxy)carbamate	2.371	57.062	5,350,271	3,136,768,594	0.17%	1,991,656	4,733,100	C9H19NO3	91426-13-0	813	874	SemiStandardNonPolar	mainlib	CK
1-Penten-3-one	2.377	55.072	17,020,088	3,136,768,594	0.54%	5,694,161	9,297,801	C5H8O	1629-58-9	790	802	SemiStandardNonPolar	mainlib	CK
Pentanal	2.471	44.055	9,033,306	3,136,768,594	0.29%	3,480,920	11,039,592	C5H10O	110-62-3	822	858	SemiStandardNonPolar	mainlib	CK
Butanoic acid, methyl ester	2.756	43.044	57,136,841	3,136,768,594	1.82%	24,499,964	109,986,055	C5H10O2	623-42-7	955	956	SemiStandardNonPolar	mainlib	CK
2-Pentenal, (E)-	3.28	55.072	2,498,350	3,136,768,594	0.08%	481,665	1,778,029	C5H8O	1576-87-0	848	848	SemiStandardNonPolar	mainlib	CK
1,3,5-Cycloheptatriene	3.474	91.076	29,830,121	3,136,768,594	0.95%	9,230,975	22,270,668	C7H8	544-25-2	905	917	SemiStandardNonPolar	mainlib	CK
Acetic acid, methyl ester	3.648	74.02	6,258,728	3,136,768,594	0.20%	2,709,715	7,687,169	C3H6O2	79-20-9	701	733	SemiStandardNonPolar	mainlib	CK
Tetrahydropyran Z-10-dodecenoate	3.655	84.949	4,656,403	3,136,768,594	0.15%	1,604,620	6,953,261	C17H30O3		833	954	SemiStandardNonPolar	mainlib	CK
Butanoic acid, 2-methyl-, methyl ester	3.659	57.062	11,981,988	3,136,768,594	0.38%	4,110,840	15,056,888	C6H12O2	868-57-5	818	819	SemiStandardNonPolar	mainlib	CK
2-Ethyl-3-vinyloxirane	4.115	69.077	11,003,919	3,136,768,594	0.35%	4,576,862	12,232,144	C6H10O	34485-78-4	745	800	SemiStandardNonPolar	mainlib	CK
Hexanal	4.118	44.055	253,167,285	3,136,768,594	8.07%	100,648,259	620,389,646	C6H12O	66-25-1	911	911	SemiStandardNonPolar	mainlib	CK
Thiirane, 2,3-dimethyl-, trans-	4.192	88.092	1,585,067	3,136,768,594	0.05%	392,133	1,026,424	C4H8S	5955-98-6	718	836	SemiStandardNonPolar	mainlib	CK
Cyclotrisiloxane, hexamethyl-	4.762	207.071	752,229	3,136,768,594	0.02%	178,370	313,095	C6H18O3Si3	541-05-9	878	878	SemiStandardNonPolar	mainlib	CK
Valeric anhydride	5.235	57.062	1,330,496	3,136,768,594	0.04%	291,429	911,516	C10H18O3	2082-59-9	836	850	SemiStandardNonPolar	mainlib	CK
anti-2-Acetoxyacetaldoxime	5.54	43.044	2,799,797	3,136,768,594	0.09%	816,078	1,438,606	C4H7NO3	37858-07-4	825	844	SemiStandardNonPolar	mainlib	CK
2-Hexenal	5.547	41.077	9,243,596	3,136,768,594	0.29%	2,054,386	13,409,546	C6H10O	505-57-7	850	850	SemiStandardNonPolar	mainlib	CK
Pentanoic acid, 2-methyl-	5.744	74.02	588,187	3,136,768,594	0.02%	340,280	743,108	C6H12O2	97-61-0	722	819	SemiStandardNonPolar	mainlib	CK
Dimethyl ether	5.818	45.065	4,230,068	3,136,768,594	0.13%	696,124	1,175,717	C2H6O	115-10-6	734	858	SemiStandardNonPolar	mainlib	CK
L-Proline, 5-oxo-, n-propyl ester	5.825	84.108	36,942,506	3,136,768,594	1.18%	7,277,463	10,124,336	C8H13NO3		661	749	SemiStandardNonPolar	mainlib	CK
Hexanoic acid, 2-methyl-	5.889	74.02	2,590,811	3,136,768,594	0.08%	842,303	1,340,031	C7H14O2	4536-23-6	787	787	SemiStandardNonPolar	mainlib	CK
Pyridine, 2-chloro-6-(2-furanylmethoxy)-4-(trichloromethyl)-	5.962	81.112	1,945,813	3,136,768,594	0.06%	316,505	335,174	C11H7Cl4NO2	70166-48-2	714	744	SemiStandardNonPolar	mainlib	CK
Cyclopentene, 3-(2-methylpropyl)-	5.969	67.047	5,876,463	3,136,768,594	0.19%	1,096,912	1,939,651	C9H16	37689-12-6	735	742	SemiStandardNonPolar	mainlib	CK
Ethylbenzene	6.147	91.076	1,762,858	3,136,768,594	0.06%	397,453	588,648	C8H10	100-41-4	948	948	SemiStandardNonPolar	mainlib	CK
1,6-Diazabicyclo[4.1.0]heptane	6.345	97.062	1,488,414	3,136,768,594	0.05%	509,284	1,258,442	C5H10N2	59204-83-0	763	977	SemiStandardNonPolar	mainlib	CK
trans-2-Hexenol	6.362	57.062	92,259,735	3,136,768,594	2.94%	30,367,473	101,595,852	C6H12O		884	903	SemiStandardNonPolar	mainlib	CK
Oxalic acid, diallyl ester	6.462	41.077	27,095,146	3,136,768,594	0.86%	6,951,218	13,683,896	C8H10O4		721	816	SemiStandardNonPolar	mainlib	CK
Cyclopropane, propyl-	6.465	56.104	42,962,932	3,136,768,594	1.37%	10,633,321	32,680,537	C6H12	2415-72-7	884	895	SemiStandardNonPolar	mainlib	CK
1-Butanol, 3-methyl-, acetate	6.818	43.044	6,409,355	3,136,768,594	0.20%	2,001,745	5,619,575	C7H14O2	123-92-2	827	827	SemiStandardNonPolar	mainlib	CK
1-Pentyn-3-ol, 3-methyl-	6.898	69.077	752,482	3,136,768,594	0.02%	272,832	590,848	C6H10O	77-75-8	731	731	SemiStandardNonPolar	mainlib	CK
1-Butanol, 2-methyl-, acetate	6.908	43.044	15,850,582	3,136,768,594	0.51%	4,036,894	7,941,204	C7H14O2	624-41-9	782	785	SemiStandardNonPolar	mainlib	CK
p-Xylene	7.371	91.076	2,053,924	3,136,768,594	0.07%	404,029	1,396,190	C8H10	106-42-3	791	839	SemiStandardNonPolar	mainlib	CK
Heptanal	7.72	41.077	4,008,547	3,136,768,594	0.13%	1,100,180	9,053,170	C7H14O	111-71-7	924	924	SemiStandardNonPolar	mainlib	CK
Hexanoic acid, methyl ester	8.608	74.02	594,638,247	3,136,768,594	18.96%	209,731,528	712,087,595	C7H14O2	106-70-7	930	930	SemiStandardNonPolar	mainlib	CK
Hexanal, 3,3-dimethyl-	9.41	69.077	1,136,406	3,136,768,594	0.04%	312,152	1,017,130	C8H16O	55320-57-5	752	773	SemiStandardNonPolar	mainlib	CK
2-Heptenal, (Z)-	9.735	41.077	5,834,694	3,136,768,594	0.19%	1,602,962	12,007,480	C7H12O	57266-86-1	918	935	SemiStandardNonPolar	mainlib	CK
Benzaldehyde	9.873	77.061	2,373,260	3,136,768,594	0.08%	281,828	1,048,677	C7H6O	100-52-7	919	919	SemiStandardNonPolar	mainlib	CK
1-Hepten-3-one	10.533	55.072	8,347,160	3,136,768,594	0.27%	1,761,348	4,809,816	C7H12O	2918-13-0	811	821	SemiStandardNonPolar	mainlib	CK
2-Butanone, 3,3-dimethyl-1-thiocyanato-	10.584	57.062	3,952,645	3,136,768,594	0.13%	942,511	1,601,979	C7H11NOS	57518-71-5	786	889	SemiStandardNonPolar	mainlib	CK
2,6-Octadiene, (E,E)-	10.725	55.072	3,214,193	3,136,768,594	0.10%	871,321	1,097,719	C8H14	18152-31-3	678	722	SemiStandardNonPolar	mainlib	CK
n-Caproic acid vinyl ester	10.735	43.044	13,091,160	3,136,768,594	0.42%	3,357,207	7,194,748	C8H14O2	3050-69-9	864	869	SemiStandardNonPolar	mainlib	CK
Butane, 2-methoxy-2-methyl-	10.795	73.067	6,468,682	3,136,768,594	0.21%	948,284	1,672,954	C6H14O	994-05-8	657	660	SemiStandardNonPolar	mainlib	CK
Hexanal, 2,2-dimethyl-	10.805	43.044	1,868,196	3,136,768,594	0.06%	1,084,891	4,022,134	C8H16O	996-12-3	696	750	SemiStandardNonPolar	mainlib	CK
5-Hepten-2-one, 6-methyl-	10.835	108.155	1,254,214	3,136,768,594	0.04%	282,839	859,632	C8H14O	110-93-0	743	749	SemiStandardNonPolar	mainlib	CK
Furan, 2-pentyl-	10.966	81.112	6,422,847	3,136,768,594	0.20%	1,509,274	2,920,191	C9H14O	3777-69-3	848	849	SemiStandardNonPolar	mainlib	CK
Hexanoic acid, ethyl ester	11.248	88.092	10,193,464	3,136,768,594	0.32%	3,080,408	19,622,236	C8H16O2	123-66-0	918	920	SemiStandardNonPolar	mainlib	CK
Cyclotetrasiloxane, octamethyl-	11.318	281.071	10,866,787	3,136,768,594	0.35%	3,880,327	36,434,657	C8H24O4Si4	556-67-2	701	825	SemiStandardNonPolar	mainlib	CK
3-Hexen-1-ol, acetate, (E)-	11.482	43.044	34,788,463	3,136,768,594	1.11%	9,403,290	32,320,793	C8H14O2	3681-82-1	945	945	SemiStandardNonPolar	mainlib	CK
Benzene, 1,4-dichloro-	11.556	145.993	2,052,556	3,136,768,594	0.07%	512,251	1,534,564	C6H4Cl2	106-46-7	784	800	SemiStandardNonPolar	mainlib	CK
1,3-Butadiene, 2-(bromomethyl)-	11.67	145.993	768,287	3,136,768,594	0.02%	244,661	517,173	C5H7Br	23691-13-6	717	883	SemiStandardNonPolar	mainlib	CK
Cyclopentene, 1-methyl-	11.677	67.047	5,835,726	3,136,768,594	0.19%	2,175,629	5,438,968	C6H10	693-89-0	709	741	SemiStandardNonPolar	mainlib	CK
Acetic acid, hexyl ester	11.687	43.044	238,634,832	3,136,768,594	7.61%	93,220,548	303,592,603	C8H16O2	142-92-7	905	905	SemiStandardNonPolar	mainlib	CK
2-Hexen-1-ol, acetate, (Z)-	11.778	43.044	771,471,583	3,136,768,594	24.59%	283,951,144	948,479,475	C8H14O2	56922-75-9	939	939	SemiStandardNonPolar	mainlib	CK
Heptanoic acid, methyl ester	12.066	74.02	1,048,028	3,136,768,594	0.03%	288,113	568,249	C8H16O2	106-73-0	861	861	SemiStandardNonPolar	mainlib	CK
D-Limonene	12.133	68.101	435,094	3,136,768,594	0.01%	80,817	298,883	C10H16	5989-27-5	841	841	SemiStandardNonPolar	mainlib	CK
2-Octenal, (E)-	13.038	41.077	5,066,176	3,136,768,594	0.16%	1,770,348	14,822,833	C8H14O	2548-87-0	889	889	SemiStandardNonPolar	mainlib	CK
Cyclobutanone, 2,2,3-trimethyl-	13.116	55.072	3,891,715	3,136,768,594	0.12%	1,069,823	3,734,900	C7H12O	1449-49-6	695	783	SemiStandardNonPolar	mainlib	CK
3(2H)-Furanone, 4-methoxy-2,5-dimethyl-	13.122	43.044	9,714,031	3,136,768,594	0.31%	3,173,638	10,830,918	C7H10O3	4077-47-8	836	840	SemiStandardNonPolar	mainlib	CK
2-Methylthiolane, S,S-dioxide	13.481	55.072	624,655	3,136,768,594	0.02%	176,139	508,179	C5H10O2S	1003-46-9	703	711	SemiStandardNonPolar	mainlib	CK
Benzoic acid, methyl ester	14.145	105.078	3,032,955	3,136,768,594	0.10%	615,039	1,549,722	C8H8O2	93-58-3	896	898	SemiStandardNonPolar	mainlib	CK
Linalool	14.266	71.043	19,296,332	3,136,768,594	0.62%	7,244,344	63,720,248	C10H18O	78-70-6	918	919	SemiStandardNonPolar	mainlib	CK
Silane, cyclohexyldimethoxymethyl-	14.38	105.078	721,018	3,136,768,594	0.02%	176,185	311,929	C9H20O2Si	17865-32-6	688	716	SemiStandardNonPolar	mainlib	CK
Nonanal	14.39	57.062	9,679,142	3,136,768,594	0.31%	3,140,696	28,253,366	C9H18O	124-19-6	923	923	SemiStandardNonPolar	mainlib	CK
trans-2-Hexenyl propionate	14.561	57.062	1,811,496	3,136,768,594	0.06%	531,265	997,049	C9H16O2		708	744	SemiStandardNonPolar	mainlib	CK
4-Octenoic acid, methyl ester	14.745	74.02	711,797	3,136,768,594	0.02%	225,332	1,667,485	C9H16O2	1732-00-9	756	761	SemiStandardNonPolar	mainlib	CK
Octanoic acid, methyl ester	14.967	74.02	34,512,426	3,136,768,594	1.10%	11,560,045	41,147,899	C9H18O2	111-11-5	918	919	SemiStandardNonPolar	mainlib	CK
2,6-Nonadienal, (E,Z)-	15.752	41.077	900,942	3,136,768,594	0.03%	264,630	872,666	C9H14O	557-48-2	781	788	SemiStandardNonPolar	mainlib	CK
Cyclopentasiloxane, decamethyl-	15.855	73.067	77,908,751	3,136,768,594	2.48%	30,176,035	73,688,997	C10H30O5Si5	541-02-6	842	843	SemiStandardNonPolar	mainlib	CK
trans-2-Nonenal	15.906	41.077	1,701,015	3,136,768,594	0.05%	555,971	4,576,612	C9H16O		898	905	SemiStandardNonPolar	mainlib	CK
Methyl salicylate	16.828	120.034	8,866,346	3,136,768,594	0.28%	1,762,214	8,826,263	C8H8O3	119-36-8	827	862	SemiStandardNonPolar	mainlib	CK
Decanal	17.096	41.077	1,250,773	3,136,768,594	0.04%	423,304	4,082,413	C10H20O	112-31-2	880	880	SemiStandardNonPolar	mainlib	CK
2,6-Dimethylbenzaldehyde	17.361	133.068	925,851	3,136,768,594	0.03%	165,452	554,758	C9H10O	1123-56-4	759	768	SemiStandardNonPolar	mainlib	CK
Valeric anhydride	17.915	57.062	114,449	3,136,768,594	0.00%	58,643	203,302	C10H18O3	2082-59-9	926	950	SemiStandardNonPolar	mainlib	CK
2-Decenal, (Z)-	18.471	41.077	755,141	3,136,768,594	0.02%	246,046	2,312,250	C10H18O	2497-25-8	835	838	SemiStandardNonPolar	mainlib	CK
Sulfurous acid, 2-ethylhexyl hexyl ester	18.907	71.043	314,131	3,136,768,594	0.01%	117,701	523,532	C14H30O3S		774	786	SemiStandardNonPolar	mainlib	CK
Undecanoic acid, 2-methyl-	19.937	74.02	393,734	3,136,768,594	0.01%	136,862	247,713	C12H24O2	24323-25-9	770	775	SemiStandardNonPolar	mainlib	CK
Cyclohexasiloxane, dodecamethyl-	20.024	73.067	38,778,512	3,136,768,594	1.24%	15,154,943	35,504,164	C12H36O6Si6	540-97-6	762	765	SemiStandardNonPolar	mainlib	CK
Ethylene glycol Formate Isobutyrate	20.581	71.043	351,618	3,136,768,594	0.01%	117,903	221,589	C7H12O4		652	770	SemiStandardNonPolar	mainlib	CK
Propanoic acid, 2-methyl-, 3-hydroxy-2,2,4-trimethylpentyl ester	21.047	71.043	423,878	3,136,768,594	0.01%	144,998	568,661	C12H24O3	77-68-9	719	719	SemiStandardNonPolar	mainlib	CK
Hexanoic acid, 5-hexenyl ester	21.359	99.083	528,799	3,136,768,594	0.02%	169,016	817,205	C12H22O2	108058-81-7	666	677	SemiStandardNonPolar	mainlib	CK
3-Hexanone, 2,5-dimethyl-	23.663	57.062	359,423	3,136,768,594	0.01%	140,022	502,806	C8H16O	1888-57-9	740	780	SemiStandardNonPolar	mainlib	CK
3-Isopropoxy-1,1,1,7,7,7-hexamethyl-3,5,5-tris(trimethylsiloxy)tetrasiloxane	23.686	73.067	5,407,143	3,136,768,594	0.17%	1,953,261	5,088,190	C18H52O7Si7	71579-69-6	765	818	SemiStandardNonPolar	mainlib	CK
Hexanoic acid, octyl ester	25.303	117.051	341,829	3,136,768,594	0.01%	109,562	569,397	C14H28O2	4887-30-3	708	713	SemiStandardNonPolar	mainlib	CK
Propanoic acid, 2-methyl-, anhydride	25.628	71.043	1,908,727	3,136,768,594	0.06%	682,181	1,249,241	C8H14O3	97-72-3	767	926	SemiStandardNonPolar	mainlib	CK
Dibutyl phthalate	31.969	149.037	1,332,318	3,136,768,594	0.04%	418,965	608,480	C16H22O4	84-74-2	910	910	SemiStandardNonPolar	mainlib	CK

Acetaldehyde	1.385	44.071	9,640,622	3,272,623,421	0.29%	2,864,234	6,327,558	C2H4O	75-07-0	805	805	SemiStandardNonPolar	mainlib	*FaHMGR*
Butane	1.512	42.998	1,261,834	3,272,623,421	0.04%	649,086	1,796,054	C4H10	106-97-8	790	793	SemiStandardNonPolar	mainlib	*FaHMGR*
Borane-methyl sulfide complex	1.566	62.05	36,167,731	3,272,623,421	1.11%	17,531,693	64,524,157	C2H9BS	13292-87-0	930	930	SemiStandardNonPolar	mainlib	*FaHMGR*
Acetic acid, methyl ester	1.58	42.998	294,077,400	3,272,623,421	8.99%	125,607,147	179,287,826	C3H6O2	79-20-9	949	949	SemiStandardNonPolar	mainlib	*FaHMGR*
2,3-Butanedione	1.764	42.998	13,071,028	3,272,623,421	0.40%	3,109,194	3,915,474	C4H6O2	431-03-8	952	952	SemiStandardNonPolar	mainlib	*FaHMGR*
Furan, 3-methyl-	1.841	82.108	144,286	3,272,623,421	0.00%	88,401	221,706	C5H6O	930-27-8	826	840	SemiStandardNonPolar	mainlib	*FaHMGR*
Furan, 2-methyl-	1.888	42.998	6,131,133	3,272,623,421	0.19%	1,697,951	5,257,608	C5H6O	534-22-5	738	773	SemiStandardNonPolar	mainlib	*FaHMGR*
Trichloromethane	1.905	83.062	13,426,747	3,272,623,421	0.41%	6,620,296	19,675,151	CHCl3	67-66-3	870	870	SemiStandardNonPolar	mainlib	*FaHMGR*
Methyl propionate	1.985	57.061	1,770,992	3,272,623,421	0.05%	613,045	1,063,404	C4H8O2	554-12-1	942	942	SemiStandardNonPolar	mainlib	*FaHMGR*
Isopropyl acetate	2.193	42.998	1,365,948	3,272,623,421	0.04%	447,032	590,083	C5H10O2	108-21-4	741	759	SemiStandardNonPolar	mainlib	*FaHMGR*
1-Penten-3-one	2.391	55.06	10,172,389	3,272,623,421	0.31%	3,443,738	4,971,489	C5H8O	1629-58-9	818	821	SemiStandardNonPolar	mainlib	*FaHMGR*
1H-Tetrazole, 1-methyl-	2.475	55.06	6,791,622	3,272,623,421	0.21%	2,269,362	2,621,754	C2H4N4	16681-77-9	777	816	SemiStandardNonPolar	mainlib	*FaHMGR*
Pentanal	2.478	44.071	26,084,741	3,272,623,421	0.80%	10,759,553	35,526,439	C5H10O	110-62-3	882	913	SemiStandardNonPolar	mainlib	*FaHMGR*
Butanoic acid, methyl ester	2.777	42.998	16,128,228	3,272,623,421	0.49%	6,227,013	27,953,000	C5H10O2	623-42-7	932	935	SemiStandardNonPolar	mainlib	*FaHMGR*
2-Butenal, 3-methyl-	3.303	55.06	2,064,118	3,272,623,421	0.06%	367,702	1,100,854	C5H8O	107-86-8	828	830	SemiStandardNonPolar	mainlib	*FaHMGR*
Acetonitrile, (dimethylamino)-	3.478	83.062	356,630	3,272,623,421	0.01%	147,960	299,222	C4H8N2	926-64-7	824	912	SemiStandardNonPolar	mainlib	*FaHMGR*
1-Pentanol	3.484	55.06	2,141,312	3,272,623,421	0.07%	748,942	3,319,244	C5H12O	71-41-0	886	886	SemiStandardNonPolar	mainlib	*FaHMGR*
Toluene	3.494	91.083	8,965,661	3,272,623,421	0.27%	2,260,223	4,935,770	C7H8	108-88-3	926	926	SemiStandardNonPolar	mainlib	*FaHMGR*
N-Methyl methacrylamide	3.565	41.075	2,229,765	3,272,623,421	0.07%	518,960	1,194,368	C5H9NO	2/3/3887	889	905	SemiStandardNonPolar	mainlib	*FaHMGR*
Butanoic acid, 2-methyl-, methyl ester	3.682	88.077	5,763,353	3,272,623,421	0.18%	1,689,159	9,071,823	C6H12O2	868-57-5	907	907	SemiStandardNonPolar	mainlib	*FaHMGR*
Hexanal	4.132	44.071	225,447,018	3,272,623,421	6.89%	87,485,022	547,532,859	C6H12O	66-25-1	907	907	SemiStandardNonPolar	mainlib	*FaHMGR*
Cyclotrisiloxane, hexamethyl-	4.772	207.052	1,228,304	3,272,623,421	0.04%	295,647	571,012	C6H18O3Si3	541-05-9	935	935	SemiStandardNonPolar	mainlib	*FaHMGR*
1,3-Dimethyldiaziridine	5.564	42.998	1,729,546	3,272,623,421	0.05%	520,584	2,011,263	C3H8N2	26177-36-6	769	837	SemiStandardNonPolar	mainlib	*FaHMGR*
2-Hexenal	5.567	41.075	848,613,180	3,272,623,421	25.93%	178,171,211	939,487,340	C6H10O	505-57-7	814	814	SemiStandardNonPolar	mainlib	*FaHMGR*
2-Pentyn-4-one	5.973	67.055	7,479,348	3,272,623,421	0.23%	1,330,004	2,013,814	C5H6O	7299-55-0	742	909	SemiStandardNonPolar	mainlib	*FaHMGR*
Ethylbenzene	6.157	91.083	3,225,287	3,272,623,421	0.10%	698,741	1,351,721	C8H10	100-41-4	855	855	SemiStandardNonPolar	mainlib	*FaHMGR*
1,6-Diazabicyclo[4.1.0]heptane	6.352	97.068	2,126,255	3,272,623,421	0.06%	540,022	914,250	C5H10N2	59204-83-0	919	974	SemiStandardNonPolar	mainlib	*FaHMGR*
trans-2-Hexenol	6.368	57.061	98,249,149	3,272,623,421	3.00%	32,663,168	109,460,871	C6H12O		889	906	SemiStandardNonPolar	mainlib	*FaHMGR*
Oxalic acid, diallyl ester	6.469	41.075	32,831,998	3,272,623,421	1.00%	7,837,244	15,285,797	C8H10O4		755	838	SemiStandardNonPolar	mainlib	*FaHMGR*
Formic acid, hexyl ester	6.476	56.09	45,595,720	3,272,623,421	1.39%	11,428,666	35,243,625	C7H14O2	629-33-4	783	783	SemiStandardNonPolar	mainlib	*FaHMGR*
L-Proline, propyl ester	6.855	70.087	528,238	3,272,623,421	0.02%	143,026	201,618	C8H15NO2		674	788	SemiStandardNonPolar	mainlib	*FaHMGR*
1,2,5-Oxadiazole	6.949	42.998	1,606,431	3,272,623,421	0.05%	391,664	682,540	C2H2N2O	288-37-9	814	973	SemiStandardNonPolar	mainlib	*FaHMGR*
Benzene, 1,3-dimethyl-	7.381	91.083	2,702,093	3,272,623,421	0.08%	519,785	1,462,958	C8H10	108-38-3	853	853	SemiStandardNonPolar	mainlib	*FaHMGR*
Heptanal	7.733	41.075	3,153,010	3,272,623,421	0.10%	828,933	6,810,586	C7H14O	111-71-7	866	868	SemiStandardNonPolar	mainlib	*FaHMGR*
Isobutyronitrile	8.612	68.105	1,432,356	3,272,623,421	0.04%	500,784	1,148,665	C4H7N	78-82-0	681	895	SemiStandardNonPolar	mainlib	*FaHMGR*
Hexanoic acid, methyl ester	8.619	74.042	205,332,658	3,272,623,421	6.27%	67,468,828	237,665,819	C7H14O2	106-70-7	940	940	SemiStandardNonPolar	mainlib	*FaHMGR*
1-Butene, 3,3-dimethyl-	9.42	69.067	1,102,015	3,272,623,421	0.03%	293,445	1,147,606	C6H12	558-37-2	658	723	SemiStandardNonPolar	mainlib	*FaHMGR*
2-Heptenal, (Z)-	9.742	41.075	7,343,993	3,272,623,421	0.22%	1,601,572	11,419,747	C7H12O	57266-86-1	925	932	SemiStandardNonPolar	mainlib	*FaHMGR*
Benzaldehyde	9.89	77.051	2,156,058	3,272,623,421	0.07%	251,819	808,714	C7H6O	100-52-7	881	881	SemiStandardNonPolar	mainlib	*FaHMGR*
2-[(Trimethylsilyl)oxy]-2-{4-[(trimethylsilyl)oxy]phenyl}ethanamine	10.121	267.018	585,081	3,272,623,421	0.02%	184,749	491,906	C14H27NO2Si2		758	775	SemiStandardNonPolar	mainlib	*FaHMGR*
1-Octen-3-one	10.537	55.06	10,682,587	3,272,623,421	0.33%	2,037,478	6,248,689	C8H14O	4312-99-6	816	839	SemiStandardNonPolar	mainlib	*FaHMGR*
1-Octen-3-ol	10.587	57.061	6,189,322	3,272,623,421	0.19%	1,149,732	1,860,108	C8H16O	3391-86-4	861	862	SemiStandardNonPolar	mainlib	*FaHMGR*
n-Caproic acid vinyl ester	10.735	42.998	20,068,756	3,272,623,421	0.61%	6,848,051	13,296,530	C8H14O2	3050-69-9	840	840	SemiStandardNonPolar	mainlib	*FaHMGR*
Butanal, 2-ethyl-	10.819	42.998	9,488,795	3,272,623,421	0.29%	2,144,553	5,223,311	C6H12O	97-96-1	738	772	SemiStandardNonPolar	mainlib	*FaHMGR*
Furan, 2-pentyl-	10.976	81.129	3,349,679	3,272,623,421	0.10%	773,266	1,394,639	C9H14O	3777-69-3	816	818	SemiStandardNonPolar	mainlib	*FaHMGR*
Cyclohexene, 4-bromo-	11.181	81.129	1,669,609	3,272,623,421	0.05%	323,927	562,581	C6H9Br	3540-84-9	809	853	SemiStandardNonPolar	mainlib	*FaHMGR*
Pentanoic acid, 3-methyl-, ethyl ester	11.275	88.077	1,797,127	3,272,623,421	0.05%	439,073	2,163,429	C8H16O2	5870-68-8	764	764	SemiStandardNonPolar	mainlib	*FaHMGR*
Cyclotetrasiloxane, octamethyl-	11.322	281.057	21,568,529	3,272,623,421	0.66%	7,734,191	30,147,085	C8H24O4Si4	556-67-2	837	868	SemiStandardNonPolar	mainlib	*FaHMGR*
Octanal	11.332	41.075	4,362,650	3,272,623,421	0.13%	1,362,097	9,387,985	C8H16O	124-13-0	758	759	SemiStandardNonPolar	mainlib	*FaHMGR*
3-Hexen-1-ol, acetate, (Z)-	11.489	67.055	29,061,474	3,272,623,421	0.89%	7,757,156	26,984,851	C8H14O2	3681-71-8	951	951	SemiStandardNonPolar	mainlib	*FaHMGR*
Benzene, 1,2-dichloro-	11.566	146.009	1,209,345	3,272,623,421	0.04%	291,060	912,089	C6H4Cl2	95-50-1	904	904	SemiStandardNonPolar	mainlib	*FaHMGR*
Acetic acid, hexyl ester	11.691	42.998	188,907,463	3,272,623,421	5.77%	74,397,820	241,243,003	C8H16O2	142-92-7	924	924	SemiStandardNonPolar	mainlib	*FaHMGR*
2-(2-Chloroethyl)-1-methylpyrrolidine	11.764	84.113	4,763,292	3,272,623,421	0.15%	1,355,000	1,646,279	C7H14ClN		657	680	SemiStandardNonPolar	mainlib	*FaHMGR*
2-Hexen-1-ol, acetate, (Z)-	11.781	42.998	779,875,046	3,272,623,421	23.83%	285,463,039	960,584,430	C8H14O2	56922-75-9	944	944	SemiStandardNonPolar	mainlib	*FaHMGR*
Heptanoic acid, methyl ester	12.073	74.042	829,799	3,272,623,421	0.03%	203,079	381,226	C8H16O2	106-73-0	846	846	SemiStandardNonPolar	mainlib	*FaHMGR*
Bicyclo[3.1.0]hex-2-ene, 4-methyl-1-(1-methylethyl)-	12.14	93.085	293,025	3,272,623,421	0.01%	81,338	248,088	C10H16	28634-89-1	794	798	SemiStandardNonPolar	mainlib	*FaHMGR*
2-Octenal, (E)-	13.045	41.075	5,111,818	3,272,623,421	0.16%	1,411,308	12,321,524	C8H14O	2548-87-0	888	890	SemiStandardNonPolar	mainlib	*FaHMGR*
3(2H)-Furanone, 4-methoxy-2,5-dimethyl-	13.156	42.998	1,111,874	3,272,623,421	0.03%	381,995	805,893	C7H10O3	4077-47-8	678	680	SemiStandardNonPolar	mainlib	*FaHMGR*
Cyclobutane, butyl-	13.481	56.09	531,299	3,272,623,421	0.02%	142,971	768,839	C8H16	13152-44-8	820	820	SemiStandardNonPolar	mainlib	*FaHMGR*
Linalool	14.276	71.074	4,397,373	3,272,623,421	0.13%	1,536,163	1,335,8054	C10H18O	78-70-6	889	889	SemiStandardNonPolar	mainlib	*FaHMGR*
Nonanal	14.397	57.061	5,958,147	3,272,623,421	0.18%	1,808,660	16,244,932	C9H18O	124-19-6	918	918	SemiStandardNonPolar	mainlib	*FaHMGR*
Octanoic acid, methyl ester	14.974	74.042	17,452,985	3,272,623,421	0.53%	5,269,295	18,430,249	C9H18O2	111-11-5	902	916	SemiStandardNonPolar	mainlib	*FaHMGR*
(+)-2-Bornanone	15.51	95.068	295,928	3,272,623,421	0.01%	106,987	385,458	C10H16O	464-49-3	749	749	SemiStandardNonPolar	mainlib	*FaHMGR*
Borane, triethyl-	15.765	41.075	346,663	3,272,623,421	0.01%	137,373	349,545	C6H15B	97-94-9	710	812	SemiStandardNonPolar	mainlib	*FaHMGR*
Cyclopentasiloxane, decamethyl-	15.856	73.058	124,765,819	3,272,623,421	3.81%	48,486,461	115,043,879	C10H30O5Si5	541-02-6	784	785	SemiStandardNonPolar	mainlib	*FaHMGR*
trans-2-Nonenal	15.909	41.075	1,168,508	3,272,623,421	0.04%	376,293	2,893,682	C9H16O		851	857	SemiStandardNonPolar	mainlib	*FaHMGR*
Methyl salicylate	16.852	119.973	351,652	3,272,623,421	0.01%	101,413	394,817	C8H8O3	119-36-8	746	830	SemiStandardNonPolar	mainlib	*FaHMGR*
2-Decen-1-ol, (E)-	17.1	41.075	943,505	3,272,623,421	0.03%	325,371	2,781,129	C10H20O	18409-18-2	819	826	SemiStandardNonPolar	mainlib	*FaHMGR*
Acetic acid, octyl ester	17.268	42.998	2,431,093	3,272,623,421	0.07%	513,003	1,862,860	C10H20O2	112-14-1	815	816	SemiStandardNonPolar	mainlib	*FaHMGR*
Benzaldehyde, 2,4-dimethyl-	17.368	133.037	463,873	3,272,623,421	0.01%	115,740	483,064	C9H10O	15764-16-6	795	857	SemiStandardNonPolar	mainlib	*FaHMGR*
2-Decenal, (E)-	18.482	70.087	367,298	3,272,623,421	0.01%	95,288	628,872	C10H18O	3913-81-3	665	671	SemiStandardNonPolar	mainlib	*FaHMGR*
Octane, 6-ethyl-2-methyl-	18.904	71.074	372,613	3,272,623,421	0.01%	126,104	553,586	C11H24	62016-19-7	781	781	SemiStandardNonPolar	mainlib	*FaHMGR*
Cyclohexasiloxane, dodecamethyl-	20.024	73.058	55,392,420	3,272,623,421	1.69%	21,681,560	52,557,734	C12H36O6Si6	540-97-6	836	837	SemiStandardNonPolar	mainlib	*FaHMGR*
Propanoic acid, 2-methyl-, butyl ester	21.054	71.074	440,704	3,272,623,421	0.01%	144,964	349,289	C8H16O2	97-87-0	751	788	SemiStandardNonPolar	mainlib	*FaHMGR*
3-Decen-1-ol, acetate, (Z)-	21.366	42.998	176,161	3,272,623,421	0.01%	96,631	670,202	C12H22O2	81634-99-3	683	709	SemiStandardNonPolar	mainlib	*FaHMGR*
2,2,6,6-Tetramethylheptane	21.587	57.061	215,417	3,272,623,421	0.01%	84,943	222,383	C11H24	40117-45-1	798	808	SemiStandardNonPolar	mainlib	*FaHMGR*
Butanoic acid, 3-methyl-, octyl ester	22.415	103.008	262,868	3,272,623,421	0.01%	87,171	491,322	C13H26O2	7786-58-5	756	756	SemiStandardNonPolar	mainlib	*FaHMGR*
5,9-Undecadien-2-one, 6,10-dimethyl-	22.727	42.998	628,462	3,272,623,421	0.02%	226,763	572,169	C13H22O	689-67-8	681	681	SemiStandardNonPolar	mainlib	*FaHMGR*
Sulfurous acid, 2-ethylhexyl hexyl ester	23.666	57.061	439,001	3,272,623,421	0.01%	169,299	680,446	C14H30O3S		729	771	SemiStandardNonPolar	mainlib	*FaHMGR*
3-Isopropoxy-1,1,1,7,7,7-hexamethyl-3,5,5-tris(trimethylsiloxy)tetrasiloxane	23.686	73.058	7,937,674	3,272,623,421	0.24%	2,936,673	7,398,817	C18H52O7Si7	71579-69-6	703	746	SemiStandardNonPolar	mainlib	*FaHMGR*
Hexanoic acid, octyl ester	25.303	117.136	1,035,718	3,272,623,421	0.03%	360,493	3,382,713	C14H28O2	4887-30-3	897	898	SemiStandardNonPolar	mainlib	*FaHMGR*
2,2,4-Trimethyl-1,3-pentanediol diisobutyrate	25.628	71.074	2,266,349	3,272,623,421	0.07%	783,191	1,567,931	C16H30O4	6846-50-0	736	752	SemiStandardNonPolar	mainlib	*FaHMGR*
Hexasiloxane, tetradecamethyl-	26.943	73.058	845,626	3,272,623,421	0.03%	298,497	688,593	C14H42O5Si6	107-52-8	688	730	SemiStandardNonPolar	mainlib	*FaHMGR*
Dibutyl phthalate	31.97	149.052	2,572,797	3,272,623,421	0.08%	837,564	1,205,903	C16H22O4	84-74-2	920	926	SemiStandardNonPolar	mainlib	*FaHMGR*

Argon	1.298	40.103	6,854,964	4,137,136,347	0.17%	3,660,409	3,955,328	Ar	7440-37-1	955	999	SemiStandardNonPolar	mainlib	*FaHMGRi*
Ethanol	1.432	45.075	4,671,546	4,137,136,347	0.11%	2,086,870	3,254,898	C2H6O	64-17-5	904	904	SemiStandardNonPolar	mainlib	*FaHMGRi*
Butane	1.499	43.037	868,658	4,137,136,347	0.02%	460,370	1,424,518	C4H10	106-97-8	748	772	SemiStandardNonPolar	mainlib	*FaHMGRi*
Borane-methyl sulfide complex	1.553	62.039	25,266,163	4,137,136,347	0.61%	12,159,308	45,286,345	C2H9BS	13292-87-0	926	926	SemiStandardNonPolar	mainlib	*FaHMGRi*
Acetic acid, methyl ester	1.566	43.037	195,505,393	4,137,136,347	4.73%	82,683,604	117,572,448	C3H6O2	79-20-9	929	929	SemiStandardNonPolar	mainlib	*FaHMGRi*
Ethyl Acetate	1.871	43.037	10,377,384	4,137,136,347	0.25%	3,983,925	5,951,690	C4H8O2	141-78-6	941	941	SemiStandardNonPolar	mainlib	*FaHMGRi*
Trichloromethane	1.891	83.049	2,086,656	4,137,136,347	0.05%	1,037,122	3,787,746	CHCl3	67-66-3	856	856	SemiStandardNonPolar	mainlib	*FaHMGRi*
Methyl propionate	1.968	57.057	2,513,555	4,137,136,347	0.06%	913,728	1,631,512	C4H8O2	554-12-1	934	934	SemiStandardNonPolar	mainlib	*FaHMGRi*
Isopropyl acetate	2.176	43.037	1,330,224	4,137,136,347	0.03%	578,587	866,236	C5H10O2	108-21-4	857	857	SemiStandardNonPolar	mainlib	*FaHMGRi*
tert-Butyl N-(tert-butoxy)carbamate	2.368	57.057	2,938,539	4,137,136,347	0.07%	943,104	2,361,586	C9H19NO3	91426-13-0	774	844	SemiStandardNonPolar	mainlib	*FaHMGRi*
1-Penten-3-one	2.374	55.061	12,333,119	4,137,136,347	0.30%	4,214,306	6,913,990	C5H8O	1629-58-9	741	819	SemiStandardNonPolar	mainlib	*FaHMGRi*
1H-Tetrazole, 1-methyl-	2.461	55.061	5,645,667	4,137,136,347	0.14%	1,874,013	2,104,486	C2H4N4	16681-77-9	801	816	SemiStandardNonPolar	mainlib	*FaHMGRi*
Pentanal	2.468	44.069	15,452,421	4,137,136,347	0.37%	6,160,352	19,016,606	C5H10O	110-62-3	797	835	SemiStandardNonPolar	mainlib	*FaHMGRi*
Butanoic acid, methyl ester	2.757	43.037	43,912,458	4,137,136,347	1.06%	18,739,668	83,145,045	C5H10O2	623-42-7	963	965	SemiStandardNonPolar	mainlib	*FaHMGRi*
2-Pentenal, (E)-	3.28	55.061	1,033,203	4,137,136,347	0.02%	367,889	1,584,978	C5H8O	1576-87-0	822	822	SemiStandardNonPolar	mainlib	*FaHMGRi*
Toluene	3.484	91.067	5,103,000	4,137,136,347	0.12%	1,264,652	3,998,397	C7H8	108-88-3	814	848	SemiStandardNonPolar	mainlib	*FaHMGRi*
Bis(N-methoxy-N-methylamino)methane	3.655	74.026	3,180,557	4,137,136,347	0.08%	1,299,048	2,574,569	C5H14N2O2	6919-46-6	768	810	SemiStandardNonPolar	mainlib	*FaHMGRi*
Butanoic acid, 4-[(tetrahydro-2H-pyran-2-yl)oxy]-, methyl ester	3.662	41.068	4,706,286	4,137,136,347	0.11%	1,551,100	7,651,896	C10H18O4	93691-87-3	706	707	SemiStandardNonPolar	mainlib	*FaHMGRi*
Methyl propionate	3.665	88.074	6,374,027	4,137,136,347	0.15%	2,112,166	4,925,642	C4H8O2	554-12-1	800	800	SemiStandardNonPolar	mainlib	*FaHMGRi*
Butanoic acid, 3-hydroxy-2-([(4-methylphenyl)sulfonyl]amino)-, methyl ester	4.108	88.074	185,141	4,137,136,347	0.00%	97,115	250,846	C12H17NO5S		674	697	SemiStandardNonPolar	mainlib	*FaHMGRi*
Hexanal	4.118	44.069	372,453,605	4,137,136,347	9.00%	150,414,036	916,289,730	C6H12O	66-25-1	917	917	SemiStandardNonPolar	mainlib	*FaHMGRi*
1-(Ethanesulfonyl)-2-(ethylsulfanyl)ethane	4.185	88.074	2,406,693	4,137,136,347	0.06%	659,686	1,828,813	C6H14O2S2	42270-61-1	691	729	SemiStandardNonPolar	mainlib	*FaHMGRi*
Cyclotrisiloxane, hexamethyl-	4.765	207.037	964,866	4,137,136,347	0.02%	227,976	384,623	C6H18O3Si3	541-05-9	850	850	SemiStandardNonPolar	mainlib	*FaHMGRi*
2-Hexenal, (E)-	5.54	41.068	13,107,991	4,137,136,347	0.32%	3,015,552	22,081,879	C6H10O	6728-26-3	884	886	SemiStandardNonPolar	mainlib	*FaHMGRi*
3-Nitro-2-butanol	5.748	73.054	177,142	4,137,136,347	0.00%	99,656	207,853	C4H9NO3	6270-16-2	736	863	SemiStandardNonPolar	mainlib	*FaHMGRi*
Propene	5.842	41.068	1,362,018,994	4,137,136,347	32.92%	235,005,144	592,693,266	C3H6	115-07-1	761	826	SemiStandardNonPolar	mainlib	*FaHMGRi*
Phenol, 2-methoxy-	5.966	81.104	1,381,094	4,137,136,347	0.03%	270,670	628,005	C7H8O2	5/1/1990	881	894	SemiStandardNonPolar	mainlib	*FaHMGRi*
Difluoromethane	6.137	51.074	1,962,670	4,137,136,347	0.05%	236,123	283,406	CH2F2	10/5/1975	899	987	SemiStandardNonPolar	mainlib	*FaHMGRi*
4,6-Octadiyn-3-one, 2-methyl-	6.154	91.067	1,921,139	4,137,136,347	0.05%	420,149	697,716	C9H10O	29743-33-7	787	843	SemiStandardNonPolar	mainlib	*FaHMGRi*
1H-Pyrazole, 3-ethyl-4,5-dihydro-	6.352	69.073	10,415,350	4,137,136,347	0.25%	3,158,254	7,985,625	C5H10N2	5920-29-6	661	689	SemiStandardNonPolar	mainlib	*FaHMGRi*
trans-2-Hexenol	6.362	57.057	64,567,139	4,137,136,347	1.56%	20,259,410	62,155,133	C6H12O		843	862	SemiStandardNonPolar	mainlib	*FaHMGRi*
Methacrolein	6.466	41.068	23,145,957	4,137,136,347	0.56%	5,217,379	9,729,342	C4H6O	78-85-3	679	725	SemiStandardNonPolar	mainlib	*FaHMGRi*
Formic acid, hexyl ester	6.472	56.103	29,477,865	4,137,136,347	0.71%	7,235,936	22,895,375	C7H14O2	629-33-4	767	768	SemiStandardNonPolar	mainlib	*FaHMGRi*
Propene	6.814	41.068	1,543,804	4,137,136,347	0.04%	422,602	1,013,892	C3H6	115-07-1	812	904	SemiStandardNonPolar	mainlib	*FaHMGRi*
1-Butanol, 3-methyl-, acetate	6.828	43.037	3,731,192	4,137,136,347	0.09%	1,134,794	2,796,748	C7H14O2	123-92-2	854	854	SemiStandardNonPolar	mainlib	*FaHMGRi*
Oxalic acid, butyl cyclobutyl ester	6.908	55.061	1,994,746	4,137,136,347	0.05%	606,914	1,588,198	C10H16O4		689	717	SemiStandardNonPolar	mainlib	*FaHMGRi*
1-Butanol, 2-methyl-, acetate	6.915	43.037	8,937,826	4,137,136,347	0.22%	2,167,345	4,505,015	C7H14O2	624-41-9	856	858	SemiStandardNonPolar	mainlib	*FaHMGRi*
o-Xylene	7.368	91.067	1,977,791	4,137,136,347	0.05%	424,676	1,740,834	C8H10	95-47-6	749	860	SemiStandardNonPolar	mainlib	*FaHMGRi*
Heptanal	7.717	41.068	4,666,592	4,137,136,347	0.11%	1,359,899	10,508,931	C7H14O	111-71-7	897	897	SemiStandardNonPolar	mainlib	*FaHMGRi*
Hexanoic acid, methyl ester	8.609	74.026	401,699,989	4,137,136,347	9.71%	142,576,828	490,195,832	C7H14O2	106-70-7	924	924	SemiStandardNonPolar	mainlib	*FaHMGRi*
.alpha.-Pinene	8.89	93.088	1,264,447	4,137,136,347	0.03%	408,610	1,466,083	C10H16	80-56-8	906	908	SemiStandardNonPolar	mainlib	*FaHMGRi*
Butane	9.729	43.037	2,870,236	4,137,136,347	0.07%	856,693	1,201,136	C4H10	106-97-8	744	930	SemiStandardNonPolar	mainlib	*FaHMGRi*
2-Heptenal, (Z)-	9.732	41.068	7,001,092	4,137,136,347	0.17%	1,967,289	13,617,963	C7H12O	57266-86-1	897	906	SemiStandardNonPolar	mainlib	*FaHMGRi*
(1H)Pyrrole-2-carbonitrile, 5-methyl-	9.88	105.052	3,531,394	4,137,136,347	0.09%	361,270	787,581	C6H6N2	26173-92-2	874	876	SemiStandardNonPolar	mainlib	*FaHMGRi*
.beta.-Pinene	10.393	93.088	1,983,448	4,137,136,347	0.05%	675,761	2,492,280	C10H16	127-91-3	870	870	SemiStandardNonPolar	mainlib	*FaHMGRi*
(4-Methylphenyl) methanol, neopentyl ether	10.517	57.057	261,117	4,137,136,347	0.01%	134,612	398,100	C13H20O		695	723	SemiStandardNonPolar	mainlib	*FaHMGRi*
N-(2-Methylacryloyl)imidazola	10.52	41.068	1,808,322	4,137,136,347	0.04%	414,578	895,178	C7H8N2O	54060-80-9	652	809	SemiStandardNonPolar	mainlib	*FaHMGRi*
1-Octen-3-one	10.527	55.061	11,924,307	4,137,136,347	0.29%	2,493,083	7,011,638	C8H14O	4312-99-6	816	830	SemiStandardNonPolar	mainlib	*FaHMGRi*
Ethylenimine	10.574	43.037	2,015,902	4,137,136,347	0.05%	542,769	810,173	C2H5N	151-56-4	691	982	SemiStandardNonPolar	mainlib	*FaHMGRi*
2-Butanone, 3,3-dimethyl-1-thiocyanato-	10.584	57.057	6,454,261	4,137,136,347	0.16%	1,148,952	1,830,316	C7H11NOS	57518-71-5	835	904	SemiStandardNonPolar	mainlib	*FaHMGRi*
n-Caproic acid vinyl ester	10.728	43.037	21,920,767	4,137,136,347	0.53%	7,382,077	15,020,649	C8H14O2	3050-69-9	875	876	SemiStandardNonPolar	mainlib	*FaHMGRi*
Propanoic acid, 2-(aminooxy)-	10.765	60.04	9,339,396	4,137,136,347	0.23%	1,192,900	2,116,708	C3H7NO3	2786-22-3	875	914	SemiStandardNonPolar	mainlib	*FaHMGRi*
Butanal, 2,2-dimethyl-	10.815	43.037	8,467,481	4,137,136,347	0.20%	2,049,947	5,827,785	C6H12O	2094-75-9	814	839	SemiStandardNonPolar	mainlib	*FaHMGRi*
5-Hepten-2-one, 6-methyl-	10.829	108.129	1,637,662	4,137,136,347	0.04%	411,827	2,156,435	C8H14O	110-93-0	749	749	SemiStandardNonPolar	mainlib	*FaHMGRi*
Furan, 2-pentyl-	10.966	81.104	5,075,876	4,137,136,347	0.12%	1,295,754	2,769,505	C9H14O	3777-69-3	889	889	SemiStandardNonPolar	mainlib	*FaHMGRi*
1-Formyl-3-methylaziridine-2-carbonitrile	11.238	81.104	1,619,753	4,137,136,347	0.04%	504,659	746,429	C5H6N2O		706	740	SemiStandardNonPolar	mainlib	*FaHMGRi*
Hexanoic acid, ethyl ester	11.244	88.074	17,912,735	4,137,136,347	0.43%	5,787,620	37,541,037	C8H16O2	123-66-0	914	914	SemiStandardNonPolar	mainlib	*FaHMGRi*
trans-2-(2-Pentenyl)furan	11.295	107.106	365,757	4,137,136,347	0.01%	105,192	293,407	C9H12O	70424-14-5	859	864	SemiStandardNonPolar	mainlib	*FaHMGRi*
Butane, 2-chloro-2-methyl-	11.305	77.058	533,487	4,137,136,347	0.01%	132,375	242,239	C5H11Cl	594-36-5	734	785	SemiStandardNonPolar	mainlib	*FaHMGRi*
Cyclotetrasiloxane, octamethyl-	11.318	281.081	1,3734,977	4,137,136,347	0.33%	4,759,649	50,869,233	C8H24O4Si4	556-67-2	671	810	SemiStandardNonPolar	mainlib	*FaHMGRi*
3-Hexen-1-ol, acetate, (Z)-	11.483	67.049	32,565,006	4,137,136,347	0.79%	8,077,864	26,799,716	C8H14O2	3681-71-8	936	936	SemiStandardNonPolar	mainlib	*FaHMGRi*
Benzene, 1,2-dichloro-	11.556	145.989	2,077,673	4,137,136,347	0.05%	500,609	1,845,266	C6H4Cl2	95-50-1	907	911	SemiStandardNonPolar	mainlib	*FaHMGRi*
4-(Benzyl-ethyl-amino)-butyric acid, methyl ester	11.667	91.067	807,870	4,137,136,347	0.02%	188,537	407,799	C14H21NO2		761	877	SemiStandardNonPolar	mainlib	*FaHMGRi*
Cyclopentene, 4-methyl-	11.677	67.049	6,174,294	4,137,136,347	0.15%	2,216,338	6,107,560	C6H10	1759-81-5	750	793	SemiStandardNonPolar	mainlib	*FaHMGRi*
Acetic acid, hexyl ester	11.684	43.037	227,652,145	4,137,136,347	5.50%	89,378,323	287,956,314	C8H16O2	142-92-7	899	899	SemiStandardNonPolar	mainlib	*FaHMGRi*
2-Hexen-1-ol, acetate, (Z)-	11.778	43.037	771,430,669	4,137,136,347	18.65%	283,039,513	957,173,820	C8H14O2	56922-75-9	937	937	SemiStandardNonPolar	mainlib	*FaHMGRi*
4-Carene, (1S,3S,6R)-(-)-	12.137	93.088	408,733	4,137,136,347	0.01%	134,988	663,001	C10H16	5208-50-4	773	786	SemiStandardNonPolar	mainlib	*FaHMGRi*
.beta.-Ocimene	12.784	93.088	700,636	4,137,136,347	0.02%	161,101	695,417	C10H16	13877-91-3	837	842	SemiStandardNonPolar	mainlib	*FaHMGRi*
2-Octenal, (E)-	13.039	41.068	5,296,702	4,137,136,347	0.13%	1,877,358	16,009,923	C8H14O	2548-87-0	889	889	SemiStandardNonPolar	mainlib	*FaHMGRi*
3(2H)-Furanone, 4-methoxy-2,5-dimethyl-	13.119	43.037	10,334,397	4,137,136,347	0.25%	3,334,596	15,441,173	C7H10O3	4077-47-8	869	873	SemiStandardNonPolar	mainlib	*FaHMGRi*
1-Octanol	13.468	56.103	1,073,732	4,137,136,347	0.03%	305,913	2,006,659	C8H18O	111-87-5	885	885	SemiStandardNonPolar	mainlib	*FaHMGRi*
Hexanal, 2,2-dimethyl-	14.112	57.057	815,368	4,137,136,347	0.02%	241,735	681,650	C8H16O	996-12-3	733	780	SemiStandardNonPolar	mainlib	*FaHMGRi*
Benzoic acid, methyl ester	14.129	105.052	7,090,079	4,137,136,347	0.17%	1,610,277	4,806,991	C8H8O2	93-58-3	905	917	SemiStandardNonPolar	mainlib	*FaHMGRi*
Linalool	14.266	71.064	15,480,031	4,137,136,347	0.37%	5,768,583	50,931,632	C10H18O	78-70-6	923	924	SemiStandardNonPolar	mainlib	*FaHMGRi*
Benzoic acid, hydrazide	14.38	77.058	1,022,949	4,137,136,347	0.02%	281,012	1,067,233	C7H8N2O	613-94-5	695	885	SemiStandardNonPolar	mainlib	*FaHMGRi*
Nonanal	14.387	57.057	16,764,329	4,137,136,347	0.41%	5,690,900	50,590,576	C9H18O	124-19-6	918	918	SemiStandardNonPolar	mainlib	*FaHMGRi*
trans-2-Hexenyl propionate	14.561	57.057	2,036,927	4,137,136,347	0.05%	591,314	873,838	C9H16O2		658	668	SemiStandardNonPolar	mainlib	*FaHMGRi*
3-Octenoic acid, methyl ester, (E)-	14.749	74.026	346,279	4,137,136,347	0.01%	120,578	753,161	C9H16O2	35234-16-3	684	712	SemiStandardNonPolar	mainlib	*FaHMGRi*
Octanoic acid, methyl ester	14.97	74.026	21,760,193	4,137,136,347	0.53%	6,910,515	24,553,846	C9H18O2	111-11-5	907	910	SemiStandardNonPolar	mainlib	*FaHMGRi*
4-Hydroxymandelic acid, 3TMS derivative	14.984	267.024	273,437	4,137,136,347	0.01%	104,741	378,927	C17H32O4Si3	37148-64-4	651	695	SemiStandardNonPolar	mainlib	*FaHMGRi*
Bicyclo[2.2.1]heptan-2-one, 1,7,7-trimethyl-, (1S)-	15.497	95.059	1,748,563	4,137,136,347	0.04%	639,840	4,001,336	C10H16O	464-48-2	920	929	SemiStandardNonPolar	mainlib	*FaHMGRi*
2,6-Nonadienal, (E,Z)-	15.755	41.068	294,669	4,137,136,347	0.01%	169,269	539,930	C9H14O	557-48-2	719	722	SemiStandardNonPolar	mainlib	*FaHMGRi*
Cyclopentasiloxane, decamethyl-	15.856	73.054	121,804,008	4,137,136,347	2.94%	47,422,966	117,059,678	C10H30O5Si5	541-02-6	827	828	SemiStandardNonPolar	mainlib	*FaHMGRi*
trans-2-Nonenal	15.906	41.068	1,685,647	4,137,136,347	0.04%	528,661	4,017,979	C9H16O		905	906	SemiStandardNonPolar	mainlib	*FaHMGRi*
Benzoic acid, ethyl ester	16.231	105.052	1,859,705	4,137,136,347	0.04%	346,985	796,009	C9H10O2	93-89-0	870	870	SemiStandardNonPolar	mainlib	*FaHMGRi*
.alpha.-Terpineol	16.748	59.039	269,627	4,137,136,347	0.01%	100,389	448,396	C10H18O	98-55-5	750	752	SemiStandardNonPolar	mainlib	*FaHMGRi*
Methyl salicylate	16.821	120.039	16,134,633	4,137,136,347	0.39%	3,800,894	15,077,032	C8H8O3	119-36-8	935	936	SemiStandardNonPolar	mainlib	*FaHMGRi*
Butyric acid, crotyl ester	16.832	71.064	2,272,593	4,137,136,347	0.05%	591,643	1,720,992	C8H14O2	20279-26-9	729	758	SemiStandardNonPolar	mainlib	*FaHMGRi*
Octanoic acid, ethyl ester	16.899	88.074	613,373	4,137,136,347	0.01%	150,525	321,622	C10H20O2	106-32-1	744	744	SemiStandardNonPolar	mainlib	*FaHMGRi*
[1,2,4]Triazolo[1,5-a]pyrazine	17.073	120.039	560,930	4,137,13,6347	0.01%	125,184	213,915	C5H4N4	399-66-6	860	940	SemiStandardNonPolar	mainlib	*FaHMGRi*
Decanal	17.093	41.068	2,293,773	4,137,136,347	0.06%	758,145	8,191,070	C10H20O	112-31-2	903	904	SemiStandardNonPolar	mainlib	*FaHMGRi*
1-Heptanol, 3-methyl-	17.267	43.037	1,014,031	4,137,136,347	0.02%	310,131	801,048	C8H18O	1070-32-2	679	685	SemiStandardNonPolar	mainlib	*FaHMGRi*
2-Ethenoxy-1,7,7-trimethylbicyclo(2.2.1)heptane	17.499	81.104	125,187	4,137,136,347	0.00%	40,126	234,154	C12H20O		656	760	SemiStandardNonPolar	mainlib	*FaHMGRi*
2-Decenal, (Z)-	18.471	41.068	636,689	4,137,136,347	0.02%	233,906	2,195,009	C10H18O	2497-25-8	841	843	SemiStandardNonPolar	mainlib	*FaHMGRi*
6-(3-Methyl)butoxytetrahydro-2H-pyran	18.904	71.064	230,495	4,137,136,347	0.01%	87,921	352,977	C10H20O2		694	716	SemiStandardNonPolar	mainlib	*FaHMGRi*
Cyclohexasiloxane, dodecamethyl-	20.024	73.054	55,574,326	4,137,136,347	1.34%	21,329,539	51,408,783	C12H36O6Si6	540-97-6	792	795	SemiStandardNonPolar	mainlib	*FaHMGRi*
.alpha.-Cubebene	20.564	105.052	501,462	4,137,136,347	0.01%	186,389	952,313	C15H24	17699-14-8	854	854	SemiStandardNonPolar	mainlib	*FaHMGRi*
(1R,5R)-2-Methyl-5-((R)-6-methylhept-5-en-2-yl)bicyclo[3.1.0]hex-2-ene	21.168	119.021	182,452	4,137,136,347	0.00%	60,883	304,369	C15H24	58319-06-5	769	791	SemiStandardNonPolar	mainlib	*FaHMGRi*
2-Buten-1-one, 1-(2,6,6-trimethyl-1,3-cyclohexadien-1-yl)-, (E)-	21.315	69.073	318,341	4,137,136,347	0.01%	110,800	252,442	C13H18O	23726-93-4	825	825	SemiStandardNonPolar	mainlib	*FaHMGRi*
(-)-.beta.-Bourbonene	21.379	81.104	421,391	4,137,136,347	0.01%	127,412	414,116	C15H24	5208-59-3	727	727	SemiStandardNonPolar	mainlib	*FaHMGRi*
6-(3-Methyl)butoxytetrahydro-2H-pyran	21.584	71.064	186,091	4,137,136,347	0.00%	61,964	314,752	C10H20O2		663	667	SemiStandardNonPolar	mainlib	*FaHMGRi*
Cycloisolongifolene	21.916	91.067	164,949	4,137,136,347	0.00%	51,322	376,142	C15H24		742	743	SemiStandardNonPolar	mainlib	*FaHMGRi*
Caryophyllene	22.134	91.067	579,480	4,137,136,347	0.01%	210,394	2,226,902	C15H24	87-44-5	836	836	SemiStandardNonPolar	mainlib	*FaHMGRi*
(S,1Z,6Z)-8-Isopropyl-1-methyl-5-methylenecyclodeca-1,6-diene	22.549	91.067	383,704	4,137,136,347	0.01%	112,948	1,066,889	C15H24	317819-80-0	757	758	SemiStandardNonPolar	mainlib	*FaHMGRi*
5,9-Undecadien-2-one, 6,10-dimethyl-	22.724	43.037	968,407	4,137,136,347	0.02%	342,784	995,993	C13H22O	689-67-8	736	736	SemiStandardNonPolar	mainlib	*FaHMGRi*
3,5-Methanocyclopentapyrazole, 3,3a,4,5,6,6a-hexahydro-3a,4,4-trimethyl-	22.855	93.088	430,483	4,137,136,347	0.01%	156,947	492,451	C10H16N2	87143-58-6	775	816	SemiStandardNonPolar	mainlib	*FaHMGRi*
Spiro[5.5]undec-2-ene, 3,7,7-trimethyl-11-methylene-, (-)-	23.535	93.088	152,925	4,137,136,347	0.00%	64,003	669,362	C15H24	18431-82-8	753	753	SemiStandardNonPolar	mainlib	*FaHMGRi*
3-Hexanone, 2,5-dimethyl-	23.663	57.057	554,873	4,137,136,347	0.01%	212,232	910,972	C8H16O	1888-57-9	653	753	SemiStandardNonPolar	mainlib	*FaHMGRi*
3-Isopropoxy-1,1,1,7,7,7-hexamethyl-3,5,5-tris(trimethylsiloxy)tetrasiloxane	23.686	73.054	10,015,897	4,137,136,347	0.24%	3,687,679	9,842,576	C18H52O7Si7	71579-69-6	754	820	SemiStandardNonPolar	mainlib	*FaHMGRi*
3,3-Dimethyl-1-phenylazetidine	23.726	105.052	1,081,981	4,137,136,347	0.03%	281,038	528,043	C11H15N	22745-26-2	804	891	SemiStandardNonPolar	mainlib	*FaHMGRi*
Camphene	23.733	93.088	1,571,960	4,137,136,347	0.04%	459,934	3,390,570	C10H16	79-92-5	766	820	SemiStandardNonPolar	mainlib	*FaHMGRi*
2,4-Di-tert-butylphenol	23.928	191.013	332,078	4,137,136,347	0.01%	116,460	303,544	C14H22O	96-76-4	701	701	SemiStandardNonPolar	mainlib	*FaHMGRi*
1-Isopropyl-4,7-dimethyl-1,2,3,5,6,8a-hexahydronaphthalene	24.243	119.021	143,351	4,137,136,347	0.00%	52,359	396,899	C15H24	16729-01-4	659	672	SemiStandardNonPolar	mainlib	*FaHMGRi*
1,6,10-Dodecatrien-3-ol, 3,7,11-trimethyl-, (E)-	24.961	69.073	1,975,335	4,137,136,347	0.05%	725,784	6,612,626	C15H26O	40716-66-3	876	880	SemiStandardNonPolar	mainlib	*FaHMGRi*
Diisoamyl ether	25.299	70.091	349,180	4,137,136,347	0.01%	122,874	424,457	C10H22O	544-01-4	686	820	SemiStandardNonPolar	mainlib	*FaHMGRi*
Hexanoic acid, octyl ester	25.303	43.037	553,030	4,137,136,347	0.01%	204,162	1,166,904	C14H28O2	4887-30-3	667	668	SemiStandardNonPolar	mainlib	*FaHMGRi*
Cyclooctasiloxane, hexadecamethyl-	26.943	73.054	1,296,128	4,137,136,347	0.03%	460,311	1,054,423	C16H48O8Si8	556-68-3	708	709	SemiStandardNonPolar	mainlib	*FaHMGRi*
Methanamine, 1,1,1-trifluoro-	27.161	84.944	219,048	4,137,136,347	0.01%	63,443	200,316	CH2F3N	61165-75-1	932	999	SemiStandardNonPolar	mainlib	*FaHMGRi*
1,2-Benzenedicarboxylic acid, butyl 2-methylpropyl ester	30.434	149.036	989,797	4,137,136,347	0.02%	328,238	463,381	C16H22O4	17851-53-5	888	896	SemiStandardNonPolar	mainlib	*FaHMGRi*
Dibutyl phthalate	31.966	149.036	3,920,483	4,137,136,347	0.09%	1,292,153	1,990,497	C16H22O4	84-74-2	916	924	SemiStandardNonPolar	mainlib	*FaHMGRi*

## Data Availability

All transcriptomic raw data reported in this paper have been deposited in the National Center for Biotechnology Information sequence read archive (transcriptome sequencing accession SRA accession number: PRJNA781262).

## Supplementary Materials

The following supporting information can be downloaded at: https://www.mdpi.com/article/10.3390/foods14071199/s1, Figure S1: Comparison of the gene transcription level detected by RNA-sequencing and qRT-PCR. The green columns indicate levels detected by RNA sequencing, and the black lines indicate the qRT-PCR result. Bars represent standard deviations of the means; Figure S2: Gene dendrogram from weighted gene co-expression network analysis (WGCNA). (A) Hierarchical clustering tree illustrating gene relationships. (B) Module–sample correlation showing the strength of associations between modules and samples. (C) Analysis of the expression patterns of differentially expressed genes (DEGs) from selected typical modules; Figure S3: Kyoto Encyclopedia of Genes and Genomes (KEGG) analysis of significantly enriched differentially accumulated metabolites (DAMs) in the comparisons of HMGRR vs. HMGRC and HMGROE vs. HMGRC; Table S1: Primer used for qRT-PCR on strawberry; Table S2: CDs sequence of *FaHMGR* and *FaHMGRi*; Table S3: General overview of transcriptome data; Table S4: The GO with the most significant enrichment of DEGs; Table S5: Genes in five main module; Table S6: The metabolites in positive and negative ion mode; Table S7: The KEGG pathways with the most significant enrichment of DEGs.
