# Supplementary figures and images for: HMGR Modulates Strawberry Fruit Coloration and Aroma Through Regulating Terpenoid and Anthocyanin Pathways
Source: Foods. 2025 Mar 29;14(7):1199. doi: 10.3390/foods14071199 (PMC11988353; doi:10.3390/foods14071199)

Relative expression (qRT-PCR)

**G1**

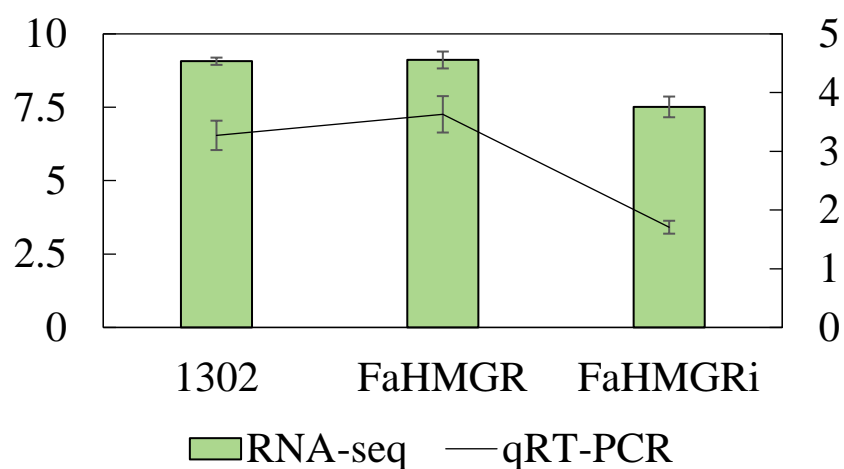

**G2**

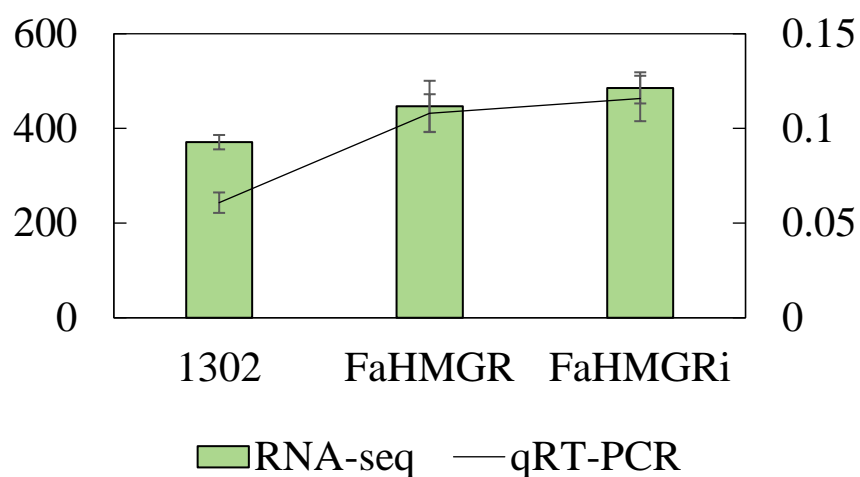

**G3**

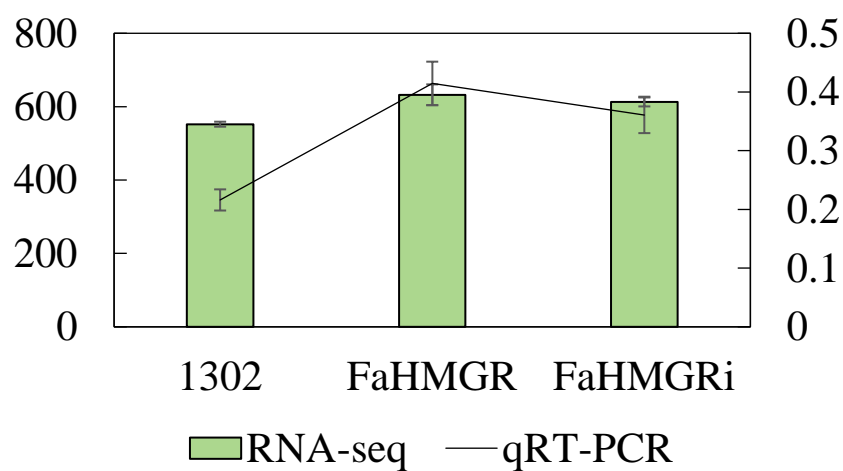

**G4**

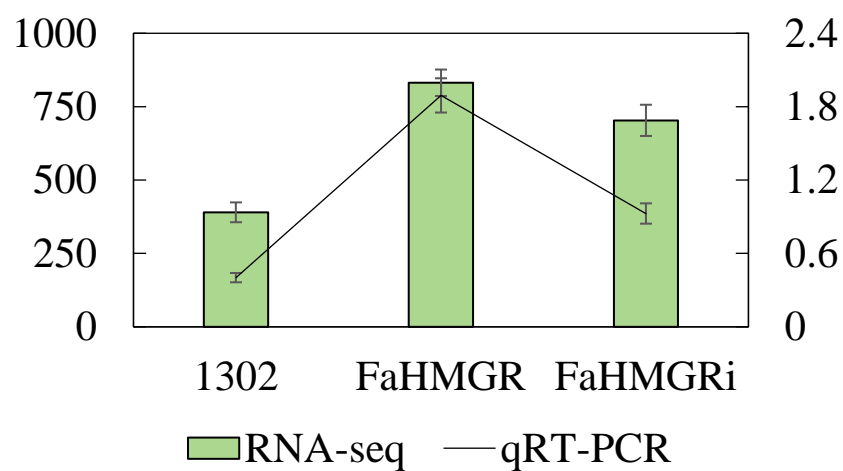

**G5**

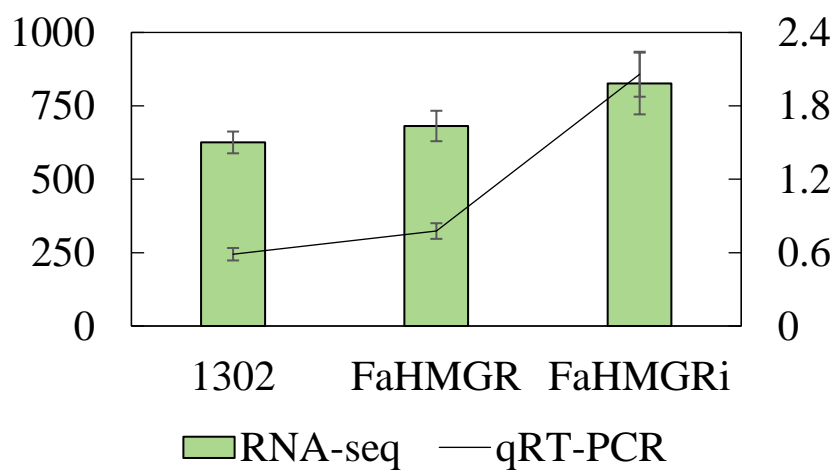

**G6**

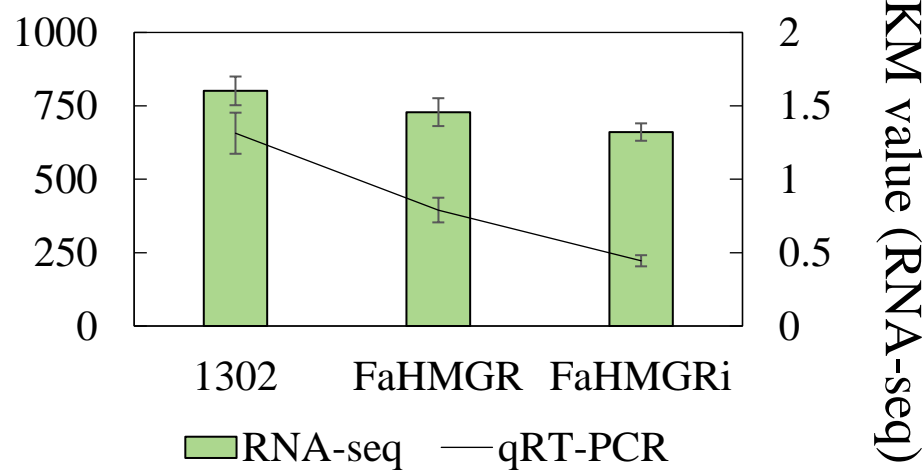

FPKM value (RNA-seq)

**G7**

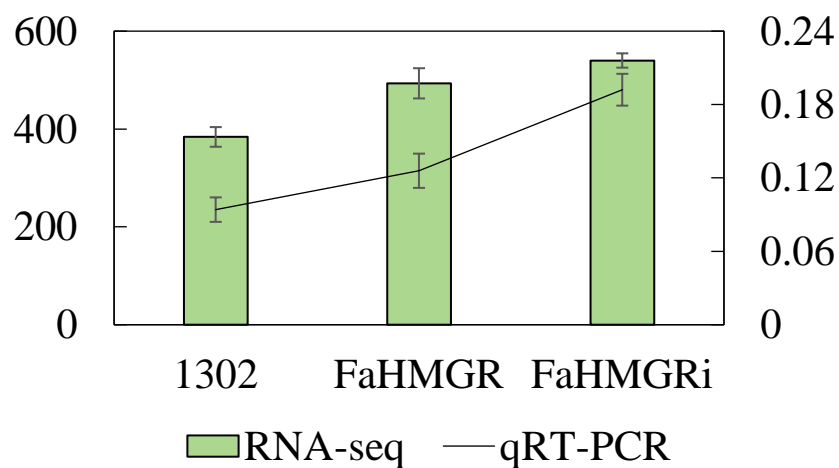

**G8**

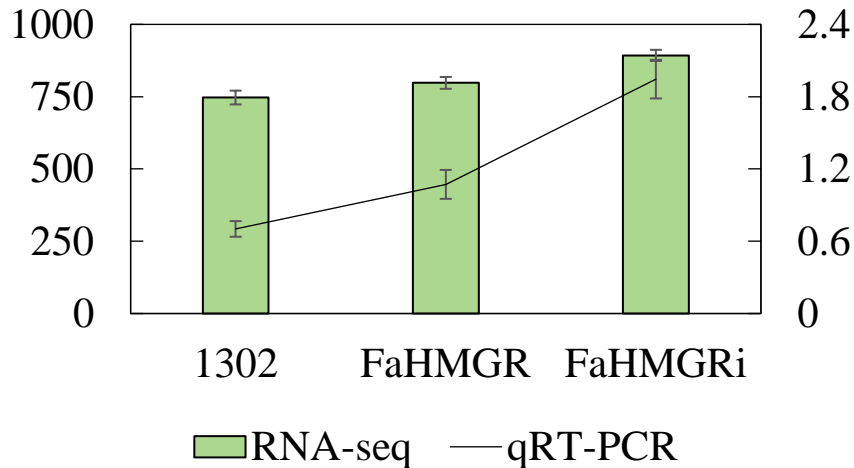

**G9**

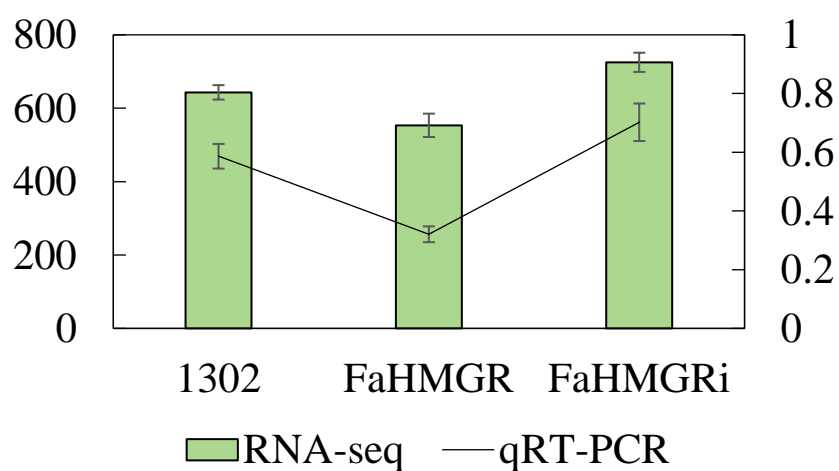

**G10**

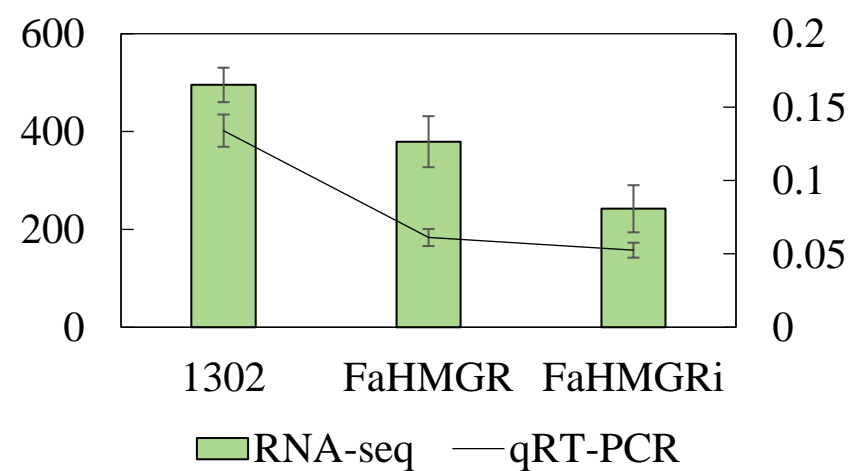

Supplement: Supplementary file 1 [file foods-14-01199-s001.zip › Figure S1.pdf]

Module-Sample relationship

A

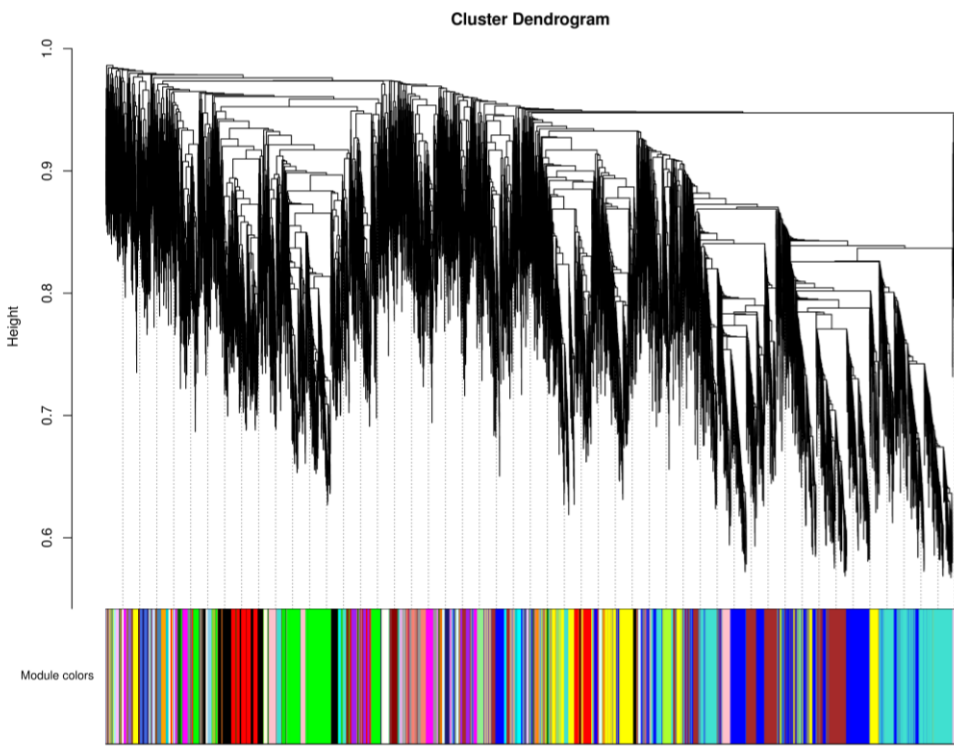

B

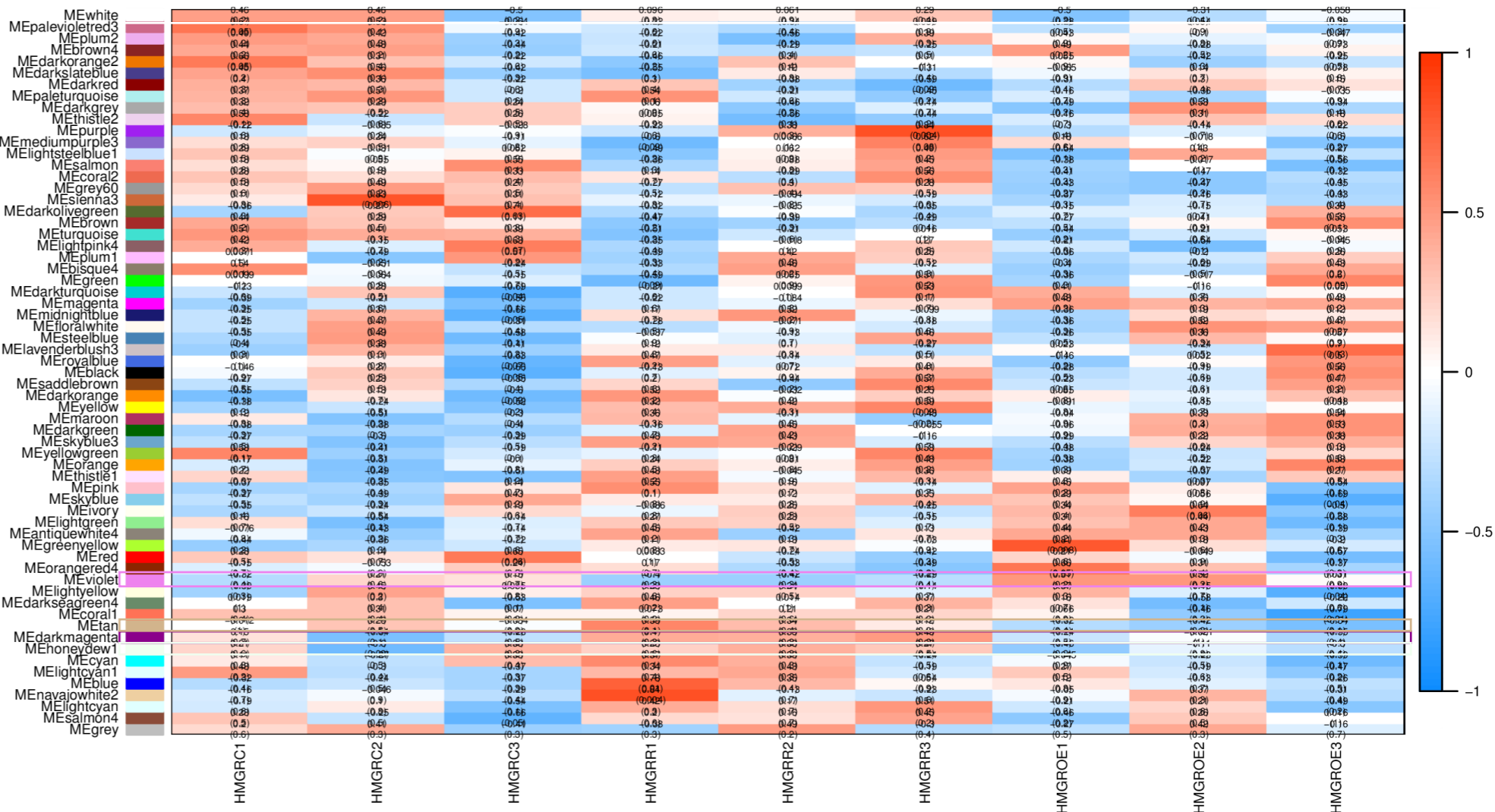

C

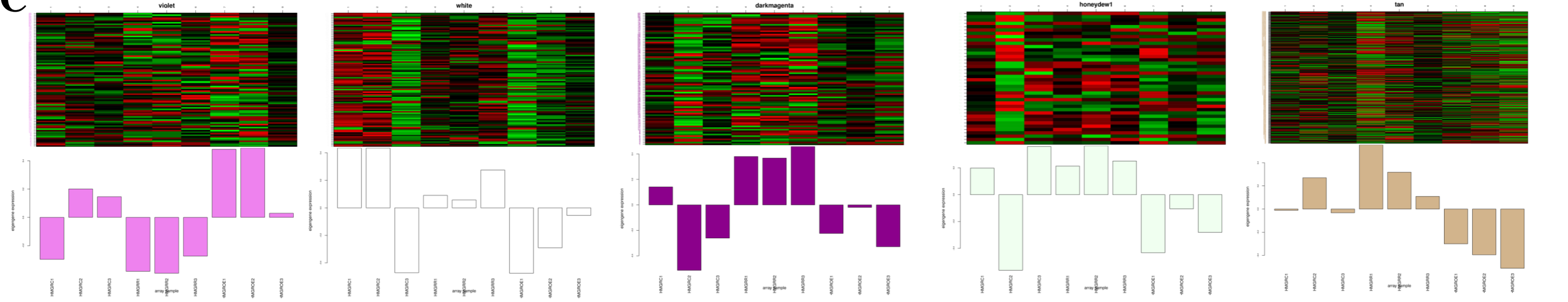

Supplement: Supplementary file 1 [file foods-14-01199-s001.zip › Figure S2.pdf]

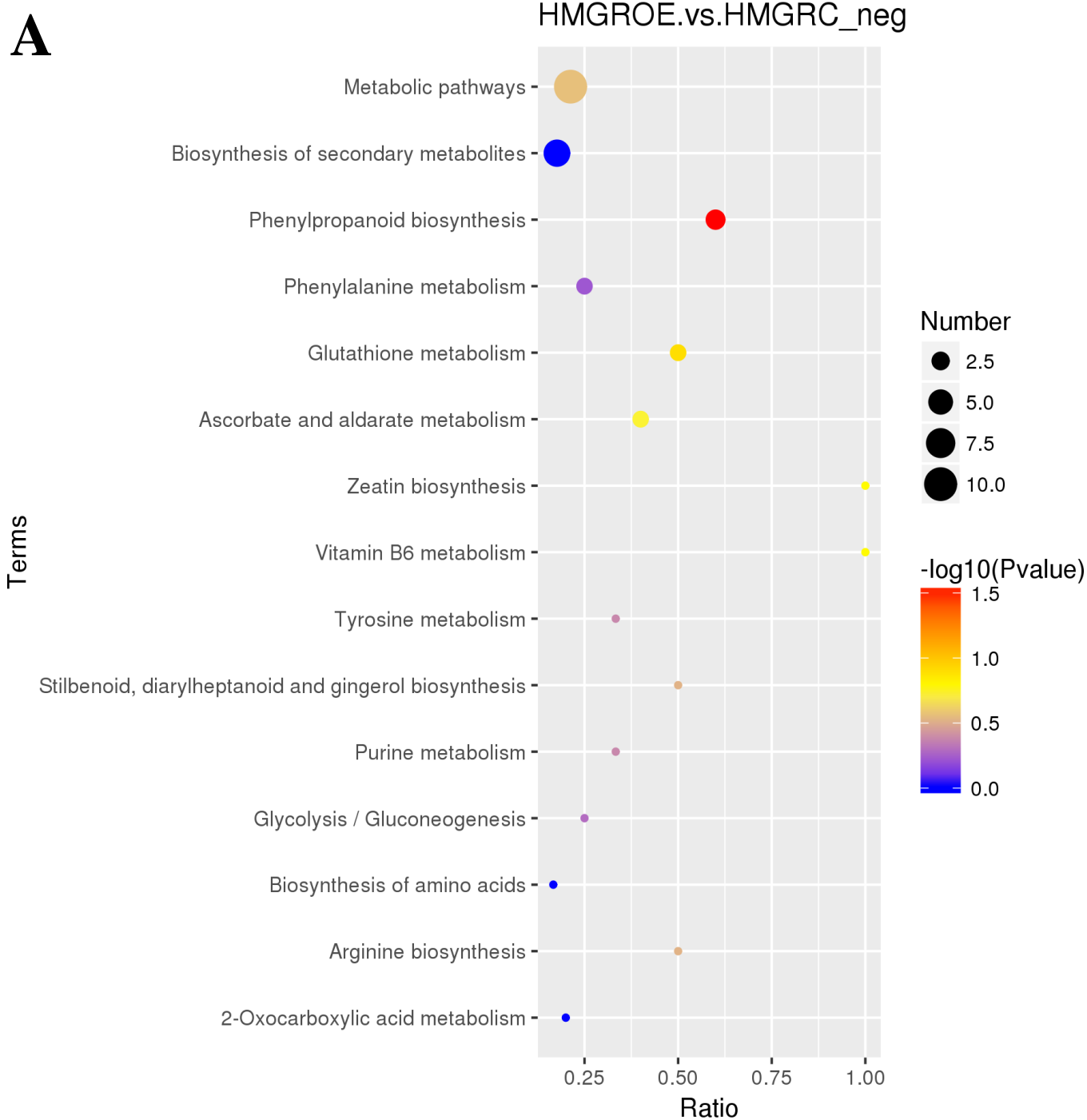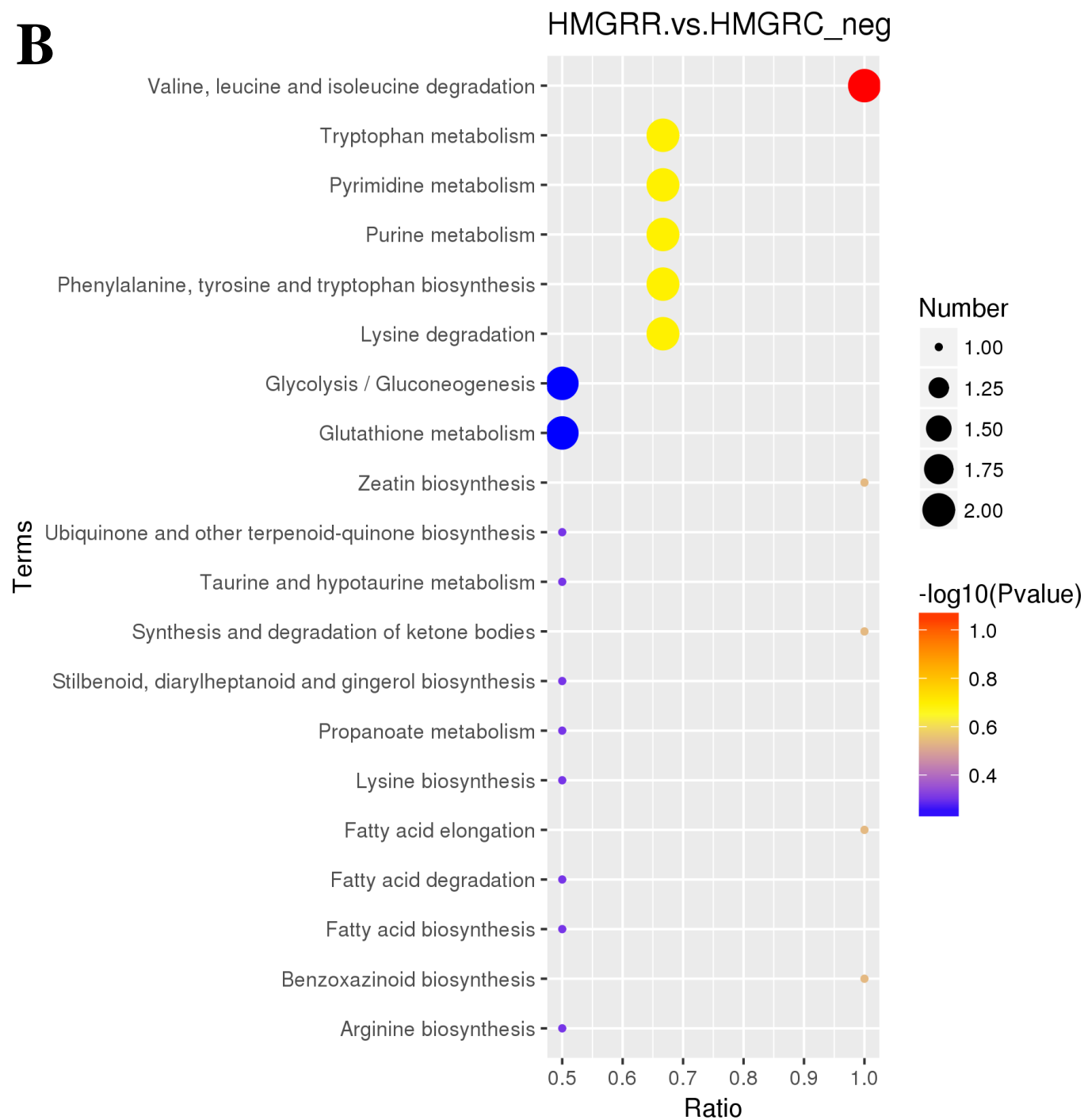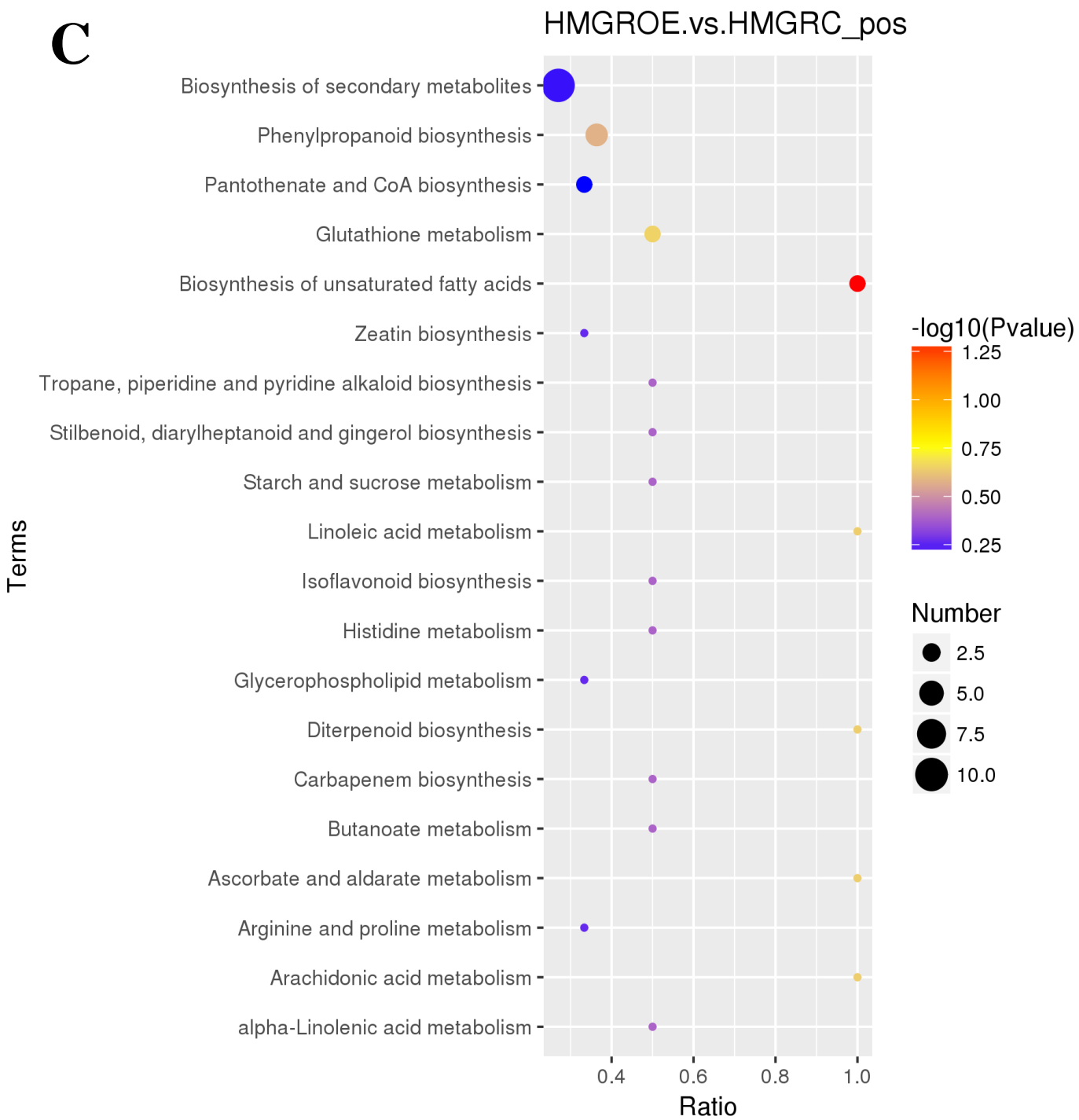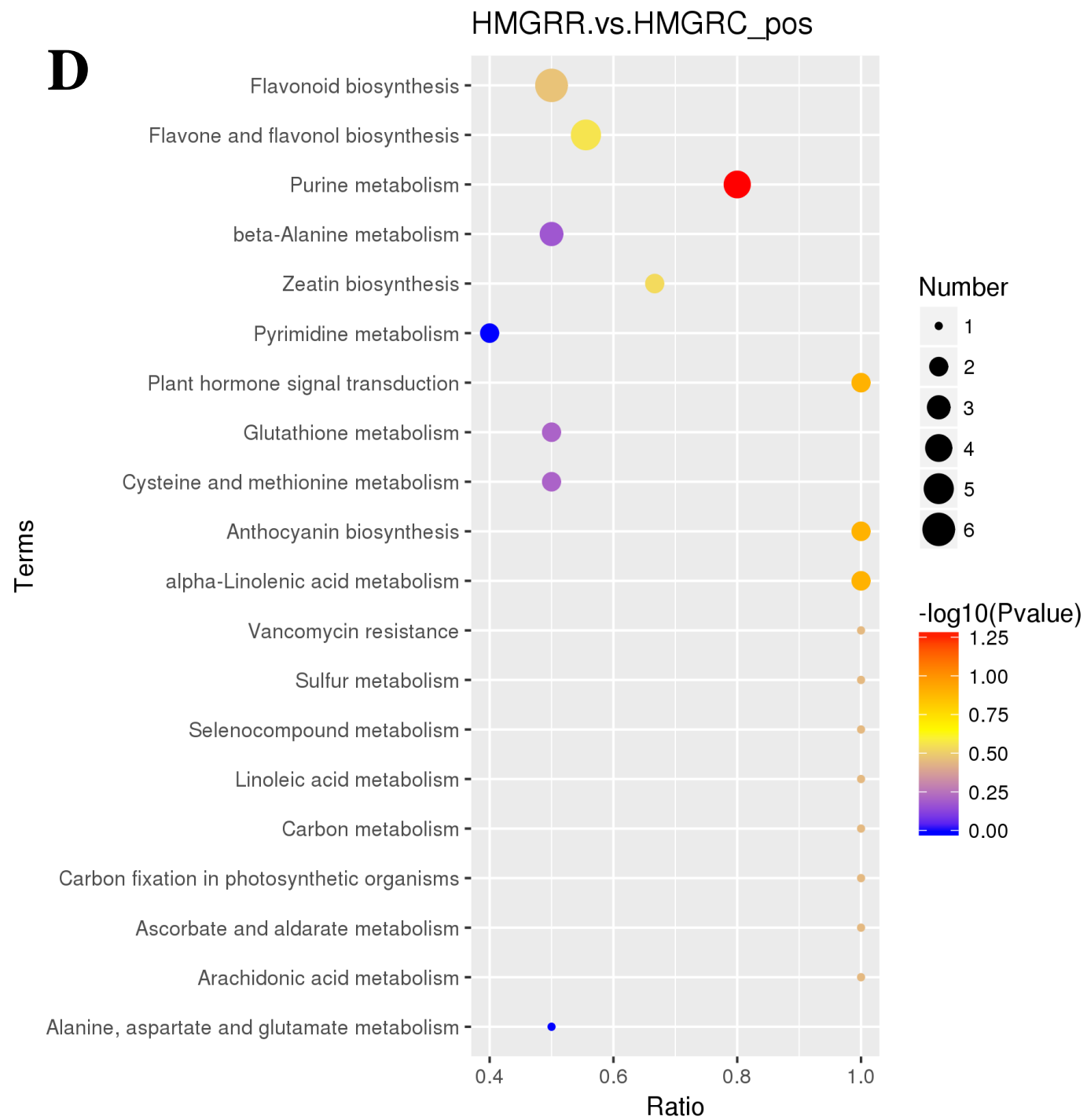

Supplement: Supplementary file 1 [file foods-14-01199-s001.zip › Figure S3.pdf]
